# Association of cigarette smoking with risk of colorectal cancer subtypes classified by gut microbiota

**DOI:** 10.18332/tid/168515

**Published:** 2023-08-01

**Authors:** Jia-An Cai, Yong-Zhen Zhang, En-Da Yu, Wei-Qun Ding, Zhao-Shen Li, Liang Zhong, Quan-Cai Cai

**Affiliations:** 1 Department of Gastroenterology and Endoscopy, Huashan Hospital, Fudan University, Shanghai, China; 2 Department of Gastroenterology, Changhai Hospital, Naval Medical University, Shanghai, China; 3 Department of Gastroenterology, 928 Hospital of PLA Joint Logistics Force, Haikou, China; 4 Department of General Surgery, Changhai Hospital, Naval Medical University, Shanghai, China; 5 National Clinical Research Center for Digestive Diseases, Shanghai, China

**Keywords:** colorectal cancer, cigarette smoking, case-control study, gut microbiota

## Abstract

**INTRODUCTION:**

Both cigarette smoking and gut microbiota play important roles in colorectal carcinogenesis. We explored whether the association between smoking and colorectal cancer (CRC) risk varies by gut microbial enterotypes and how smoking-related enterotypes promote colorectal carcinogenesis.

**METHODS:**

A case-control study was conducted. Fecal microbiota was determined by 16S rDNA sequencing. The cases with CRC or adenoma were subclassified by gut microbiota enterotypes. Multivariate analyses were used to test associations between smoking and the odds of colorectal neoplasm subtypes. Mann-Whitney U tests were used to find differential genera, genes, and pathways between the subtypes.

**RESULTS:**

Included in the study were 130 CRC patients (type I: n=77; type II: n=53), 120 adenoma patients (type I: n=66; type II: n=54), and 130 healthy participants. Smoking increased the odds for type II tumors significantly (all p for trend <0.05) but not for type I tumors. The associations of smoking with increased odds of colorectal neoplasm significantly differed by gut microbiota enterotypes (p<0.05 for heterogeneity). An increase in carcinogenic bacteria (genus *Escherichia shigella*) and a decrease in probiotics (family *Lachnospiraceae* and *Ruminococcaceae*) in type II tumors may drive disease progression by upregulating oncogenic signaling pathways and inflammatory/oxidative stress response pathways, as well as protein phospholipase D1/2, cytochrome C, and prostaglandin-endoperoxide synthase 2 expression.

**CONCLUSIONS:**

Smoking was associated with a higher odds of type II colorectal neoplasms but not type I tumors, supporting a potential role for the gut microbiota in mediating the association between smoking and colorectal neoplasms.

## INTRODUCTION

Cigarette smoking is associated with a modest but significantly increased risk of colorectal neoplasms, including colorectal cancer (CRC) and adenoma^1^. Recent studies have confirmed that smoking raises the risk of colorectal neoplasms in a dose-dependent manner^2^. Although mechanisms underlying these smoking-colorectal neoplasm associations remain unclear, it is postulated that the gut microbiota may play a mediating role^3^. Accumulating evidence suggests that gut microbiota plays an important role in colorectal carcinogenesis, with different gut microbiota compositions having different effects on the development of CRC^4-7^. For example, significant enrichment of *Fusobacterium nucleatum*^4^, *Bilophila wadsworthia*^5^, and *Escherichia coli*^4^ have been identified as potential pathogens in CRC patients, whereas probiotics including *Eubacterium*^6^ and *Faecalibacterium*^7^ have shown a protective effect against CRC.

Previous studies indicate that cigarette smoking can affect the composition and function of the gut microbiota^3,8,9^. Compared with non-smokers, a lower abundance of *Bifidobacterium* and a higher abundance of *Negativicutes* have been found in current smokers^8^. In addition, mouse models have shown that smoking can further alter metabolic processes *in vivo*, including primary bile acid biosynthesis^9^. Since enterotypes can be used to describe gut microbiota composition and function^10^, it is not surprising that there are different enterotypes between smokers and non-smokers^11^. Besides, evidence also indicates that the magnitude of the association between smoking and CRC risk differs by tumor subtypes^12^. CRC heterogeneity can be partially attributed to the variety and compositional differences of the gut microbiota and their interactions with humans^13^.

Based on the above reports, we hypothesized that the association between smoking and colorectal neoplasm risk could differ by gut microbial enterotypes. To test this hypothesis, the CRC group and adenoma group were individually split into two enterotypes (referred to as subgroups) by employing the Dirichlet multinomial mixture model according to their gut microbiota composition. Then, we further evaluated the heterogeneity between tumor subgroups concerning smoking. In order to understand how smoking-related enterotypes promote colorectal carcinogenesis, we compared the diversity, composition, and function of the gut microbiota between diverse tumor subtypes.

## METHODS

### Study population

A case-control study design was applied. From 15 July 2015 to 31 July 2016, we enrolled Han Chinese aged ≥40 years who came to Changhai Hospital (Shanghai, China) for a diagnosing colonoscopy or a screening colonoscopy as part of a routine health check-up. Individuals who received any antibiotics in the past 6 months or had an incomplete colonoscopy were excluded. Other exclusion criteria were pregnant women; a history of colorectal neoplasm, inflammatory bowel disease, hereditary polyposis syndromes, or other cancer of any type; a family history of CRC in the first- or second-degree relatives; a family history of colorectal adenoma or familial hereditary syndrome, including familial adenomatous polyposis, hereditary nonpolyposis colorectal cancer, Turcot syndrome, Oldfield syndrome, and juvenile polyposis syndrome, in the first-degree relatives younger than 60 years; and prior chemotherapy, radiotherapy, or colonic surgery. According to colonoscopic and histological findings, patients with CRC or adenoma were selected as the CRC group or the adenoma group, respectively, and individuals with no remarkable colonoscopic findings were defined as healthy controls. Individuals with multiple neoplasms were categorized according to the most histologically advanced lesion. Informed consent was obtained from all participants. The study protocol was approved by the Ethics Committee of Changhai Hospital, Naval Medical University, Shanghai, China.

### Study procedures

All eligible participants first completed a questionnaire, then provided fasting blood samples and fresh stool samples, and finally underwent colonoscopy.

A self-reported questionnaire covered information on potential risk factors, including age, sex, body mass index (BMI, kg/m^2^), smoking, drinking, and other factors as described previously^14^. Smoking was defined as smoking one or more cigarettes per day for at least one year, while the smoking index was reported as the number of packs per day multiplied by the number of smoking years (pack-years). Drinking was defined as drinking alcohol of any type more than once every week and lasting for more than 1 year.

Fasting venous blood samples (2 mL) were collected into ethylenediaminetetraacetic acid (EDTA)-containing tube and then were centrifuged at 3000 rpm for 10 min for the supernatant. Plasma endotoxin, soluble tumor necrosis factor receptor 2 (sTNFR-II), C-reactive protein (CRP), and interleukin-6 (IL-6) levels were measured by the ELISA method (Human Endotoxin ELISA Kit 96 T, Anogen; Human sTNFR-II ELISA Kit 96 T, Raybiotech; Human CRP ELISA Kit 96 T, Anogen; Human IL-6 ELISA Kit 96 T, Anogen) according to the manufacturer’s protocol.

Fresh stool samples (≥1 g) were collected and immediately placed into a -80^o^C refrigerator for storage and later use. DNA was extracted from frozen stool samples with an OMEGA-soil DNA Isolation Kit (USA Omega Bio-Tek). DNA quantification and amplification were conducted as described previously^15^. The 16S rDNA sequencing was finally conducted on the Illumina MiSeq platform (Illumina, USA).

The colonoscopy examination procedure has been detailed in our previous study^14^. In brief, polyethylene glycol lavage solution was used for bowel preparation, and colonoscopy was performed by experienced endoscopists using a standard video colonoscope (Olympus Optical Co., Tokyo, Japan). All polyps and suspicious lesions removed or biopsied during colonoscopy were sent for histologic examination.

### Taxonomic profiling

Computational biologic software, including Trimmomatic (version 0.27) and FLASH (Fast Length Adjustment of Short reads) were used for 16S rDNA sequencing data optimization and statistics^16^. According to the overlap relationships, the paired-end reads are spliced into a sequence. The quality of reads and the effect of merging are then filtered by quality control. Usearch (version 7.1) was used for operational taxonomic unit (I) cluster analysis. Based on the Silva database (Release 123)^17^, QIIME and RDP Classifier Bayesian algorithms (confidence threshold = 0.7) were used to analyze the representative sequences of OTUs with 97% similarity by taxonomy^18^. The community composition of each sample was counted at each level (domain, phylum, class, order, family, genus) to obtain the classification information corresponding to each OTU.

### Statistical analysis

IBM SPSS Statistics for Windows (version 26.0) and R for Windows (version 4.1.2) were used for all statistical analyses. A two-sided p<0.05 indicated statistical significance.

*Relationship between smoking and odds of colorectal neoplasms*

In the two case-control studies (CRC case-control and adenoma case-control studies), we performed a univariate analysis with the chi-squared test for categorical variables and the unpaired *t*-test for continuous variables comparing each case group with the control group to test the associations of each potential risk factor with colorectal neoplasms. Binary logistic backward stepwise regression analyses were then used to investigate the association between smoking and colorectal neoplasms after controlling for other potential risk factors with p<0.10 in the univariate analyses.

*Relationship between smoking and odds of colorectal neoplasm subtypes*

The CRC and adenoma cases were separately clustered into two enterotypes (referred to as subgroups or subtypes hereafter, i.e. type I and type II CRC, or type I and type II adenoma) by the Dirichlet multinomial mixture model using the R package ‘DirichletMultinomial’^19^. In the two case-case-control studies, similar univariate and multivariate analyses were used to test associations between smoking and the odds of colorectal neoplasm subtypes as described above. As part of our primary hypothesis testing, heterogeneity between the subtypes in relation to smoking was assessed by using a Wald test^20^. If there was significant heterogeneity in the relationship between the odds of different subtypes and smoking status, to further understand the reasons for the heterogeneity and how smoking-related enterotypes promote colorectal carcinogenesis, we further compared the differences in clinical characteristics, gut microbiota diversity, composition, and function between different subgroups of CRC and between different subgroups of adenoma, respectively.

*Comparisons of clinical characteristics and gut microbiota composition between subgroups*

We performed comparative analyses of clinical characteristics between subgroups using similar univariate analysis methods as described above.

Alpha-diversity, including the microbial abundance indexes Chao and ACE, and the microbial diversity indexes Shannon and Simpson, was analyzed between subgroups of CRC or adenoma group separately by Mothur (version 1.30.1)^21^. Principal coordinates analysis (PCoA) was used to visualize beta-diversity in the subgroups based on Bray-Curtis distances, and the permutational multivariate analysis of variance (PERMANOVA) using distance matrices was applied to further confirm the significant differences in microbial communities with other confounding factors (sex, age) under control.

To explore the change in gut microbiota composition, Mann-Whitney U tests were used to find differential genera between subgroups of CRC or adenoma groups in abundance with: 1) adjusted p<0.05 [false discovery rate (FDR) corrected], and 2) fold change (FC) >1.5 for significance. Pairwise correlations between the top 30 selected genera were calculated by Spearman’s correlation test and were visualized using Cytoscape (version 3.9.1) for a microbe-microbe network. We further computed the correlations between the differential genera and plasma factors in smoking-related enterotypes of each case group to preliminarily explain whether the changes in bacterial abundance are related to the role of inflammation and intestinal barrier function. In addition, to explore the association between smoking and differential microbiota, we performed partial correlation analyses adjusting for potential confounders.

To identify potential microbiota markers that differentiate between the two subtypes of colorectal cancer (CRC) and colorectal adenoma, we constructed classification models based on the top 30 different genera using two different methods, linear support vector machine and logistic regression, respectively (Supplementary file Methods).

*Gut microbiota functional analysis between subgroups*

To further explore the effects of functional changes in gut microbiota on colorectal neoplasm progression, the R package ‘Tax4FUN’ was used for functional prediction to obtain KO gene abundance and pathway abundance information based on KEGG^22^. Differential genes and pathways were selected using the same statistical methods as described above. Correlations between differential genera, genes, and pathways were analyzed by Spearman’s correlation test.

## RESULTS

The CRC group with 130 patients, the adenoma group with 120 patients, and the healthy control group with 130 participants were finally confirmed for inclusion in this study. The characteristics of all participants are shown in Table 1. After multivariable adjustment, the association of smoking with colorectal neoplasm risk remained significant (CRC: adjusted odds ratio, AOR=1.87; 95% CI: 1.05–3.35, p=0.034; adenoma: AOR=2.52; 95% CI: 1.42–4.46, p=0.002) (Table 2).

**Table 1:** Characteristics of the study population

*Characteristics*	*Colorectal cancer*			*Colorectal adenoma*			*Healthy control*
	*Type I*	*Type II*	*All*	*Type I*	*Type II*	*All*	
Total, n	77	53	130	66	54	120	130
**ACE**							
Mean (SD)	60.05 (9.98)	61.25 (9.69)	60.54 (9.84)	58.44 (9.59)	59.81 (10.75)	59.06 (10.11)	58.58 (8.88)
Range	41–88	40–80	40–88	41–83	40–84	40–84	41 – 79
**Sex**, n (%)							
Female	35 (45.5)	30 (56.6)	65 (50.0)	26 (39.4)	23 (42.6)	49 (40.8)	65 (50.0)
Male	42 (54.5)	23 (43.4)	65 (50.0)	40 (60.6)	31 (57.4)	71 (59.2)	65 (50.0)
**Smoking,** n (%)							
No	54 (70.1)	37 (69.8)	91 (70.0)	42 (63.6)	33 (61.1)	75 (62.5)	105 (80.8)
Yes	23 (29.9)	16 (30.2)	39 (30.0)	24 (36.4)	21 (38.9)	45 (37.5)	25 (19.2)
**Pack-years**							
Mean (SD)	8.00 (13.74)	8.92 (16.90)	8.38 (15.05)	9.36 (17.22)	8.74 (12.83)	9.08 (15.34)	5.84 (15.00)
Range	0–60	0–60	0–60	0–100	0–40	0–100	0 – 70
**Drinking,** n (%)							
No	56 (72.7)	42 (79.2)	98 (75.4)	48 (72.7)	39 (72.2)	87 (72.5)	109 (83.8)
Yes	21 (27.3)	11 (20.8)	32 (24.6)	18 (27.3)	15 (27.8)	33 (27.5)	21 (16.2)
**Body mass index** (kg/m^2^)							
Mean (SD)	23.75 (3.16)	23.37 (3.00)	23.59 (3.09)	23.95 (3.28)	24.13 (3.42)	24.03 (3.33)	23.79 (3.15)
Range	17.03–33.33	17.02–30.85	17.02–33.33	15.43–32.24	17.30–31.14	15.43–32.24	16.53–35.16

SD: standard deviation.

**Table 2:** Smoking and colorectal neoplasms risk, overall and subclassified by gut microbiota enterotypes^a^

*Group*	*Non-smokers*	*Smokers*	*p^b^*	*p^c^*	*Pack-years*			*p^d^*	*p^c^*
					*0*	*1–28*	*>28*		
**Healthy control** (N=130), n	105	25			105	13	12		
**All colorectal cancer** (N=130), n	91	39	0.044		91	20	19		
AOR (95% CI)	1 (Ref.)	1.87 (1.05–3.35)	0.034		1 (Ref.)	2.27 (0.99–5.22)	2.33 (0.98–5.56)	0.028	
**Type I colorectal cancer** (N=77), n	54	23	0.080		54	12	11		
AOR (95% CI)	1 (Ref.)	1.91 (0.89–4.09)	0.096		1 (Ref.)	1.94 (0.77–4.90)	1.88 (0.71–4.98)	0.134	
**Type II colorectal cancer** (N=53), n	37	16	0.107	0.033	37	8	8		0.028
AOR (95% CI)	1 (Ref.)	2.30 (1.16–4.55)	0.017		1 (Ref.)	3.04 (0.96–9.65)	3.49 (1.07–11.37)	0.025	
**All colorectal adenoma** (N=120), n	75	45	0.001		75	27	18		
AOR (95% CI)	1 (Ref.)	2.52 (1.42–4.46)	0.002		1 (Ref.)	3.20 (1.41–7.25)	2.17 (0.89–5.27)	0.025	
**Type I colorectal adenoma** (N=66), n	42	24	0.009		42	14	10		
AOR (95% CI)	1 (Ref.)	2.42 (1.08–5.44)	0.032		1 (Ref.)	2.70 (1.04–6.99)	2.11 (0.75–5.90)	0.080	
**Type II colorectal adenoma** (N=54), n	33	21	0.005	0.042	33	13	8		0.039
AOR (95% CI)	1 (Ref.)	3.23 (1.34–7.78)	0.009		1 (Ref.)	4.07 (1.47–11.25)	2.37 (0.77–7.30)	0.046	

AOR: adjusted odds ratio; adjusted for age, sex, drinking, and body mass index.

a Colorectal cancer group and colorectal adenoma group were divided into two enterotypes (termed subgroups, i.e. type I and type II colorectal cancer, or type I and type II colorectal adenoma) according to gut microbiota by using the Dirichlet multinomial mixture model, respectively.

b Values of p refer to the comparison between the case group and healthy control group in the univariate analysis or in the multivariate analysis.

c Values of p for the heterogeneity of the association of smoking with colorectal neoplasms defined by gut microbiota subtypes.

d Values of p were based on the linear trend test by using the multivariable logistic regression.

Using Dirichlet multinomial mixture model, CRC cases were clustered into two enterotypes based on gut microbiota composition, i.e. type I (n=77) and type II CRC cases (n=53) (Supplementary file, Figure 1A). Like CRC cases, colorectal adenoma cases were also divided into two subtypes, including type I (n=66) and type II adenoma cases (n=54) (Supplementary file, Figure 1B). The results from multivariate analysis showed that smoking significantly increased the odds of type II CRC (AOR=2.30; 95% CI: 1.16–4.55, p=0.017) and type II adenoma (AOR=3.23; 95% CI: 1.34–7.78, p=0.009) (Table 2). We observed higher odds of type II colorectal neoplasm with increasing pack-years smoked (CRC: p for trend =0.025; adenoma: p for trend =0.046), while we did not observe a significant trend in odds increase for type I colorectal neoplasm (CRC: p for trend =0.134; adenoma: p for trend =0.080) (Table 2). The associations of smoking with colorectal neoplasm significantly differed by gut microbiota enterotypes (p<0.05 for heterogeneity).

In both the CRC group and the adenoma group, the comparison of clinical characteristics between the two subtypes showed that serum carcinoembryonic antigen (CEA) and alkaline phosphatase (ALP) of type II enterotype (termed smoking-related enterotype) were significantly lower than those of type I enterotype (termed non-smoking-related enterotype) (Supplementary file Tables 1 and 2).

Compared with alpha-diversity in non-smoking-related enterotypes, alpha-diversity in smoking-related enterotypes showed a significant descent in the ACE estimator, Chao1 richness estimator, and Shannon index, and an increase in the Simpson index, indicating that community richness and diversity both declined in smoking-related enterotypes (Table 3). Significant differences between smoking-related enterotypes and non-smoking-related enterotypes were also found in PCoA (all p=0.001) (Supplementary file Figures 2 and 3) and PERMANOVA (CRC: F=5.78, R^2^=0.11, p=0.016; adenoma: F=7.24, R^2^=0.12, p =0.005).

**Table 3:** Difference in alpha diversity between smoking-related enterotypes and non-smoking-related enterotypes^a^

*Alpha diversity*	*Colorectal cancer (N=130)*		*p^b^*	*Colorectal adenoma (N=120)*		*p^b^*
	*Type I Mean (SD)*	*Type II Mean (SD)*		*Type I Mean (SD)*	*Type II Mean (SD)*	
Total, n	77	53		66	54	
ACE	364.49 (67.38)	261.00 (75.22)	<0.001	384.12 (68.19)	247.89 (51.53)	<0.001
Chao1	365.77 (67.83)	250.17 (71.48)	<0.001	380.56 (65.39)	239.31 (51.98)	<0.001
Shannon index	3.48 (0.40)	2.67 (0.74)	<0.001	3.56 (0.39)	2.70 (0.58)	<0.001
Simpson index	0.08 (0.04)	0.18 (0.16)	<0.001	0.07 (0.04)	0.17 (0.14)	<0.001

a The type I enterotypes of the CRC group and adenoma group were termed non-smoking-related enterotypes, while the type II enterotypes were termed smoking-related enterotypes.

b Values of p refer to the comparison between the type I subgroup and type II subgroup by using the Mann-Whitney U test. SD: standard deviation.

**Figure 1:**
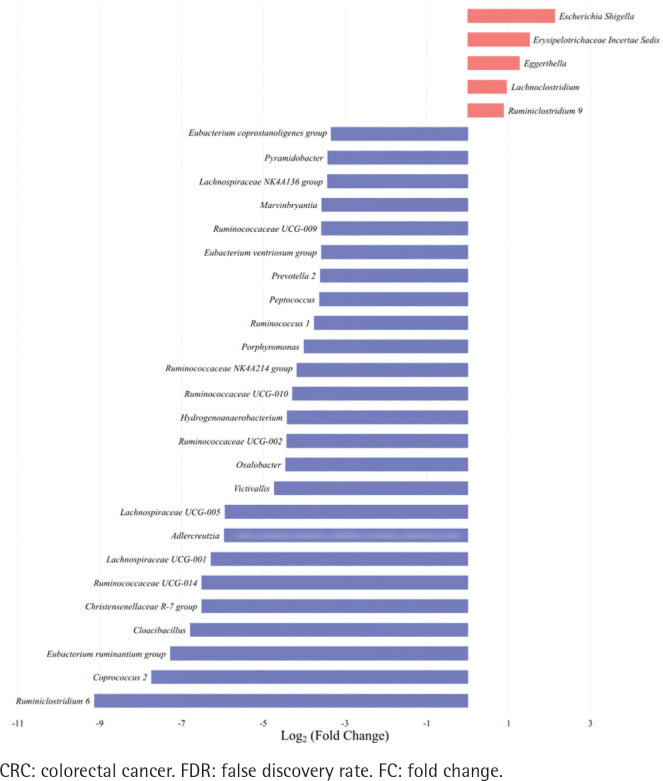
Top 30 significantly differential genera in abundance (FDR adjusted p<0.05, FC>1.5) between type II enterotype (smoking-related enterotype) and type I enterotype (non-smoking-related enterotype) within the CRC group. The bar plot shows the FC values of the top 30 differential genera in type II enterotype relative to type I enterotype. The genera are ordered by FC value from high to low. The red bars indicate increased genera abundance in type II enterotype, and the blue bars indicate decreased genera abundance in type II enterotype

**Figure 2:**
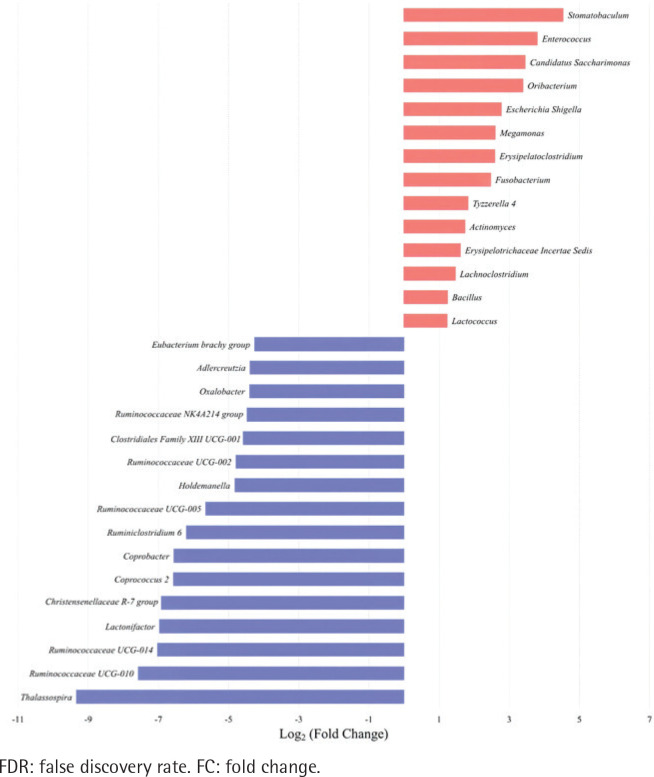
Top 30 significantly differential genera in abundance (FDR adjusted p<0.05, FC>1.5) between type II enterotype (smoking-related enterotype) and type I enterotype (non-smoking-related enterotype) within the colorectal adenoma group. The bar plot shows the FC values of the top 30 differential genera in type II enterotype relative to type I enterotype. The genera are ordered by FC value from high to low. The red bars indicate increased genera abundance in type II enterotype, and the blue bars indicate decreased genera abundance in type II enterotype

**Figure 3:**
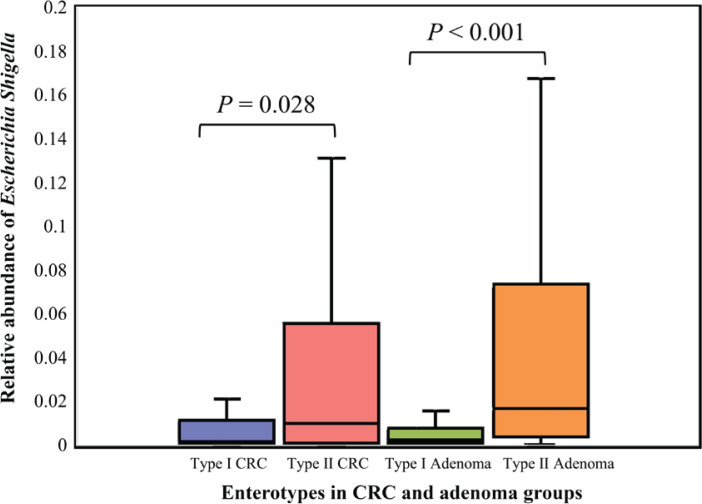
The relative abundance of Escherichia shigella in different enterotypes. The boxplots show the relative abundance of Escherichia shigella in type I and type II enterotypes of colorectal cancer and colorectal adenoma groups. Escherichia shigella was significantly more abundant in type II enterotypes than in type I enterotypes in both groups (Mann-Whitney U test, all p<0.05)

The top 30 significantly differential genera in abundance were further screened out by comparing smoking-related enterotypes with non-smoking-related enterotypes within the CRC group and adenoma group, respectively (Figures 1 and 2). Among them, there were the same increased (*Escherichia shigella*, *Erysipelotrichaceae incertae sedis*, *Lachnoclostridium*) and decreased (*Ruminiclostridium* 6, *Coprococcus* 2, *Christensenellaceae* R-7 group, *Ruminococcaceae* UCG-014, *Adlercreutzia*, *Oxalobacter*, *Ruminococcaceae* UCG-002, *Ruminococcaceae* UCG-010, *Ruminococcaceae* NK4A214 group) genera abundance in type II adenoma subgroup as in type II CRC subgroup. Of these, the pathogenic bacterium *Escherichia shigella* in the type II subgroup had the highest FC value in the elevated genera compared with the type I subgroup in the CRC group [FDR adjusted p=0.028, Log_2_(FC)=2.12], and it was also significantly more abundant in type II adenoma [FDR adjusted p<0.001, Log_2_(FC)=2.78] (Figure 3). Interestingly, we found a taxonomic chain with a significant increase in abundance from phylum to genus in both type II subgroups. This chain consisted of *Proteobacteria* (phylum), *Gammaproteobacteria* (class), *Enterobacteriales* (order), *Enterobacteriaceae* (family) and *Escherichia shigella* (genus) (Table 4).

**Table 4:** Taxonomic chain with a significant increase in abundance in the type II subgroups compared with the type I subgroups

*Taxonomy*	*Type II vs Type I in CRC group*			*Type II vs Type I in adenoma group*		
	*Log_2_ (FC)*	*p^a^*	*Adjusted p^b^*	*Log_2_ (FC)*	*p^a^*	*Adjusted p^b^*
Phylum *Proteobacteria*	1.47	0.002	0.008	1.48	0.002	0.006
Class *Gammaproteobacteria*	1.88	0.007	0.043	2.02	<0.001	<0.001
Order *Enterobacteriales*	2.09	0.003	0.036	2.13	<0.001	<0.001
Family *Enterobacteriaceae*	2.09	0.003	0.029	2.13	<0.001	<0.001
Genus *Escherichia shigella*	2.12	0.005	0.028	2.78	<0.001	<0.001

CRC: colorectal cancer. FC: fold change.

a Values of p refer to the comparison between the type I subgroup and type II subgroup by using the Mann-Whitney U test.

b Adjusted values of p were corrected by false discovery rate.

We further constructed the interaction networks of differential bacteria (Supplementary file: Figure 4 and Tables 3–6). We found that the positive and negative correlations among the differential bacteria were significantly different between smoking-related enterotypes and non-smoking-related enterotypes. For elevated pathogenic bacteria, positive correlations were observed among *Eggerthella*, *Erysipelotrichaceae incertae sedis*, and *Lachnoclostridium* in the type II CRC subgroup, and between *Bacillus* and two bacteria (*Lachnoclostridium* and *Lactococcus*) in the type II adenoma subgroup, respectively. For depleted gut-beneficial bacteria, positive correlations were observed among genera belonging to the family *Lachnospiraceae* or *Ruminococcaceae* both in type II CRC and type II adenoma subgroups. Negative correlations were observed between *Ruminococcaceae* UCG-014 and *Eggerthella* in the type II CRC subgroup, and *Tyzzerella* 4 and *Ruminococcaceae* UCG-002 in the type II adenoma subgroup, respectively, suggesting that there were antagonistic associations between the elevated pathogenic bacteria and the depleted probiotics in the smoking-related enterotypes.

**Figure 4:**
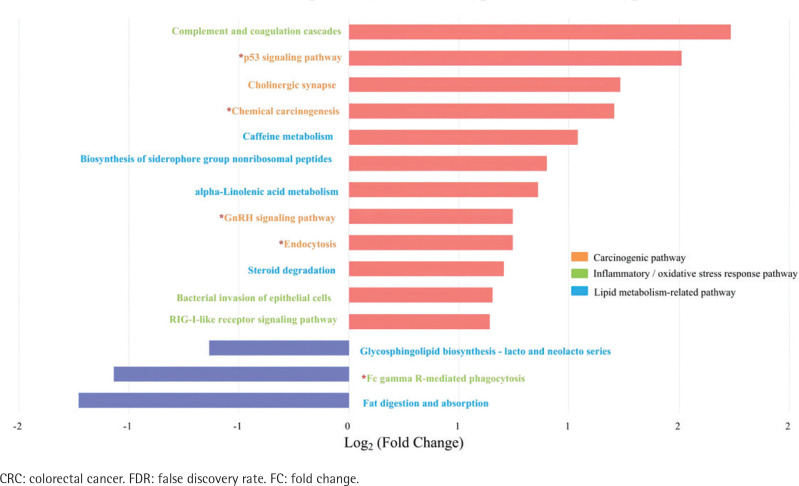
Differential pathways in type II CRC subtype relative to type I subtype based on Tax4FUN functional analysis (FDR adjusted p<0.05, FC>1.5). The bar plot shows the FC values of the differential pathways in type II CRC subtype relative to type I subtype. The pathways are ordered by FC value from high to low. The red asterisk denotes the common pathways in both type II CRC and type II adenoma

**Figure 5:**
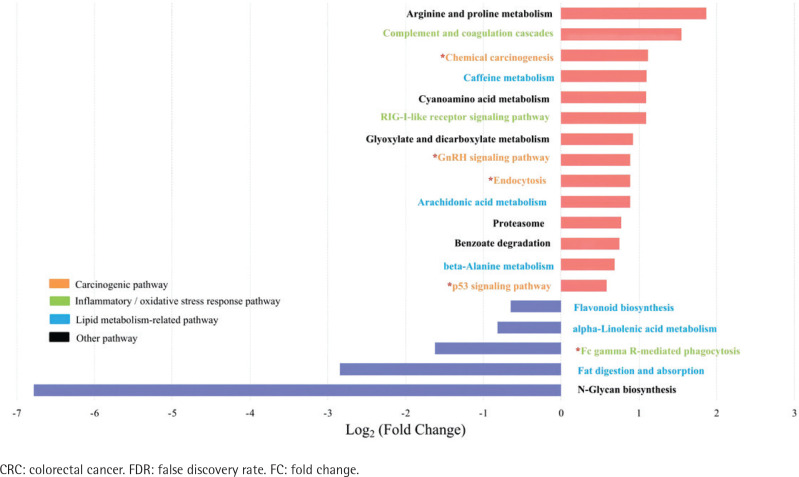
Differential pathways in type II colorectal adenoma subtype relative to type I subtype based on Tax4FUN functional analysis (FDR adjusted p<0.05, FC>1.5). The bar plot shows the FC values of the differential pathways in type II colorectal adenoma subtype relative to type I subtype. The pathways are ordered by FC value from high to low. The red asterisk denotes the common pathways in both type II CRC and type II adenoma

Spearman’s correlation analysis showed positive correlations between *Stomatobaculum* and neutrophil count and between *Tyzzerella* 4 and sTNFR-II in the type II adenoma subgroup (Supplementary file Table 7). In contrast, two depleted probiotics, including *Ruminococcaceae* UCG-014 and *Ruminococcaceae* UCG-009, were negatively correlated with sTNFR-II in the type II CRC subgroup. In addition, the *Ruminococcaceae* NK4A214 *group*, *Eubacterium coprostanoligenes group* in the type II CRC subgroup, and *Oxalobacter* in the type II adenoma subgroup were negatively correlated with endotoxin. After adjusting for age, sex, drinking, CEA, ALP, sTNFR-II, and endotoxin, partial correlation analyses showed that smoking was positively correlated with *Eggerthella* in the type II CRC subgroup and with *Family XIII* UCG-001 in the type II adenoma subgroup (Supplementary file Tables 8 and 9). There was a negative correlation with the *Christensenellaceae* R7 group in the type II CRC subgroup and with *Lactonifactor* in the type II adenoma subgroup. No significant correlation between smoking and the differential genera was observed in the type I subgroups (Supplementary file Tables 10 and 11). The results of biomarker analysis showed that linear support vector machine classifiers achieved an area under the receiver operating characteristic curve (AUC) of 0.82 and 0.86 to detect a patient with type II CRC and type II adenoma, respectively, while logistic regression classifiers achieved an AUC of 0.84 and 0.86 to detect a patient with type II CRC and type II adenoma, respectively (Supplementary file: Figures 5–8 and Tables 12 and 13). In order to further explore the role of differential bacteria in adenoma and CRC progression, Tax4FUN functional analysis was used to find specific differential pathways. Three kinds of pathways with significant changes, including carcinogenic signaling pathways, inflammatory or oxidative stress response pathways, and lipid metabolism-related pathways, were observed in type II colorectal neoplasms (Figures 4 and 5). Among the carcinogenic pathways, enrichment of the p53 signaling pathway was found in both type II CRC [FDR adjusted p=0.013, Log_2_(FC)=1.51] and type II adenoma [FDR adjusted p=0.045, Log_2_(FC)=0.59] subgroups (Supplementary file Table 14). In addition, some pathways involved in both carcinogenesis and inflammation/oxidative stress response, including chemical carcinogenesis, endocytosis, and the GnRH signaling pathway, were also enriched in both subgroups. The changes in metabolic pathways are mainly centered on lipid metabolism-related pathways, including the enrichment of steroid degradation and the decline of fat digestion and absorption.

Tax4FUN functional analysis was further used to find specific differential genes. Two kinds of genes with significant changes, including oncogenes and pro-inflammatory genes, were found in type II colorectal neoplasms (Supplementary file: Figures 9 and 10, and Table 15). Among them, gene K01115 (KEGG) encoding phospholipase D½LD) 1/2, which was found to participate in both carcinogenesis and inflammation/oxidative stress response pathways (Supplementary file Figures 11 and 12), was significantly enriched in both type II CRC [FDR adjusted p=0.016, Log_2_(FC)=1.68] and type II adenoma [FDR adjusted p=0.01, Log_2_(FC)=1.65] subgroups (Supplementary file Figure 13). We found that *Escherichia shigella* was positively correlated with PLD1/2 in not only the type II CRC subgroup but also the type II adenoma subgroup (Supplementary file: Figures 14 and 15, and Table 16).

In addition, gene K08738 (KEGG) encoding cytochrome C (CYC), which was found to participate in the p53 signaling pathway, was found to be significantly enriched in both the type II CRC [FDR adjusted p<0.001, Log_2_(FC)=1.37] and type II adenoma [FDR adjusted p=0.005, Log_2_(FC)=1.11] subgroups (Supplementary file Table 15). *Escherichia shigella* was found to positively correlate with CYC in the type II adenoma subgroup (Supplementary file Table 16). Gene K11987 (KEGG) encoding prostaglandin-endoperoxide synthase 2 (PTGS2) was also found to be significantly enriched in type II CRC with the highest increasing fold [FDR adjusted p=0.048, Log_2_(FC)=6.24] compared with type I CRC (Supplementary file Table 15). PTGS2 was primarily involved in inflammation/oxidative stress response pathways, including NF-kappa B, TNF, IL-17, and C-type lectin receptor signaling pathways (Supplementary file Figure 16). *Lachnospiraceae* NK4A136 group was negatively correlated with PTGS2 (Supplementary file Table 16).

## DISCUSSION

In this study, we found that participants with higher pack-years smoked were associated with higher odds of type II colorectal neoplasms, including type II CRC and type II adenoma, but not type I neoplasms. Our data also indicate that an increase in carcinogenic bacteria (genus *Escherichia shigella*) and a decrease in probiotics (family *Lachnospiraceae* and *Ruminococcaceae*) in type II colorectal neoplasms may drive disease progression by upregulating oncogenic signaling pathways and inflammatory/oxidative stress response pathways, as well as important proteins (PLD1/2, CYC, and PTGS2) expression encoded by related oncogenes and pro-inflammatory genes. These findings support the hypothesis that the association between smoking and the odds of colorectal neoplasm could differ by gut microbial enterotypes and thus may be mediated by modulation of specific species in the gut microbiota. To our knowledge, our study represents the first to examine the intersection of smoking and the odds of colorectal neoplasm subtypes according to gut microbiota status.

The potential role of smoking in increasing the odds of colorectal neoplasms, including CRC and colorectal adenoma, has been widely recognized^1-3,12^. However, there has been considerable heterogeneity in the epidemiological data associating smoking with the odds of colorectal neoplasms^2^. Our results suggest that the inconsistency in the association of smoking with higher CRC risk may be in part attributable to differential associations with cancer subtypes according to gut microbiota. A similar result observed in the association of smoking with the risk of adenoma, a critical precursor to CRC^23^, further confirms the effect of smoking on the development of specific CRC subtypes according to gut microbiota. Our findings are consistent with previous reports, which showed that CRC was not a single disease and smoking could selectively affect the risk of specific CRC subtypes^2,12^.

The precise mechanism by which smoking may increase the risk of type II CRC (the ‘smoking-related subtype’) remains unclear. Previous studies have demonstrated that smoking can affect the composition and function of the gut microbiota^3,8,9.^ Being consistent with the above reports, our findings showed that there was different microbial diversity, composition, and function between smoking-related and non-smoking-related subtypes and thus provided indirect evidence for the effect of smoking on gut microbiota. Specifically, different microbial alpha- and beta-diversities between tumor subtypes observed in this study indicate that the smoking-related subtype is indeed a microbial pattern differing from the non-smoking-related one. Further, we found some significantly changed genera in abundance in smoking-related subtypes, including *Escherichia shigella*, which was known for being one of the primary causes of acute colitis and diarrhea by invading epithelial cells, disrupting the intestinal barrier, and inhibiting proinflammatory cell death^24^. We also found a taxonomic chain with a significant increase in abundance from the phylum *Proteobacteria*, class *Gammaproteobacteria*, order *Enterobacteriales*, family *Enterobacteriaceae* to the genus *Escherichia shigella*, further suggesting that smoking can enhance the abundance of proinflammatory bacteria, similar to the findings by other studies^3,25.^The results from correlation analyses showed that there were antagonistic associations between the elevated pathogenic bacteria and the depleted probiotics in the smoking-related subtypes. The elevated pathogenic microbiota partly belongs to pro-inflammatory bacteria and showed positive correlations with plasma inflammatory markers. In contrast, the depleted bacteria were mainly from the short-chain fatty acids (SCFAs)-producing family *Lachnospiraceae* and *Ruminococcaceae* and showed negative correlations with plasma inflammatory marker sTNFR-II and intestinal barrier function marker endotoxin, which was commonly used to assess intestinal permeability. SCFAs are one of the end-products of gut microbiota metabolism and play an important role in lipid metabolism, energy homeostasis, regulation of the immune system, and inflammatory responses^26,27^. In addition, the partial correlation analyses were consistent with the above results. Our above findings indicate that smoking may promote colorectal carcinogenesis by increasing the abundance of pro-inflammatory bacteria and reducing the abundance of SCFAs-producing bacteria, leading to an enhanced inflammatory response and the attenuation of anti-inflammatory and intestinal barrier functions.

After that, we performed pathway enrichment analysis to further explore the molecular mechanisms of smoking-related flora changes in colorectal carcinogenesis. In smoking-related subtypes, we found three kinds of significantly changed pathways, including carcinogenic signaling pathways, inflammatory or oxidative stress response pathways, and lipid metabolism-related pathways. Specifically, our data showed that the carcinogenic pathway p53 signaling pathway, which was associated with cell cycle arrest, cellular senescence, and apoptosis and involved in the CRC development caused by smoking^28^, was enriched in the smoking-related subtypes. In addition, the GnRH signaling pathway, which was involved in both carcinogenesis and inflammatory/oxidative stress response pathways and known for its capability of activating mitogen-activated protein kinases cascades that were confirmed to participate in smoking-related carcinogenesis through an increase in abundance of *Eggerthella lenta*^3,29^, was also found to be enriched in the smoking-related subtypes.

Finally, we found significantly increased expression of oncogenes and pro-inflammatory genes in the smoking-related subgroups, which encode some important proteins, including PLD1/2, CYC, and PTGS2. Specifically, our data showed that the significantly increased expression of PLD1/2 participated in both carcinogenesis and inflammation/oxidative stress response pathways in the smoking-related subgroups. PLD isoforms have been shown to be involved in multiple stages of cancer progression^30^, such as being used by gut microbiota, including *Escherichia coli* to metabolize choline into the disease-associated metabolite trimethylamine^31^. It is also reported that PLD1 can regulate the expression of interleukin-6 in human bronchial epithelial cells induced by cigarette smoke extract^32^. There are similar findings in our study, in which a significant positive correlation was observed between elevated carcinogenic genus *Escherichia shigella* and PLD1/2 expression. According to previous reports, CYC participates in apoptosis and carcinogenesis through p53/caspases-dependent signaling pathways and *Escherichia shigella* infection can upregulate CYC expression^33,34^. Our similar results showed that the significantly increased CYC expression was positively correlated with both the p53 signaling pathway and *Escherichia shigella* abundance in the smoking-related subtypes. In addition, we also found that PTGS2, enriched in the smoking-related subtypes, was primarily involved in inflammatory/oxidative responses pathways and showed a negative correlation with the SCFAs-producing genus *Lachnospiraceae* NK4A136 group, indicating that smoking could attenuate the anti-inflammatory action and thus increase the CRC risk by reducing SCFAs-producing bacteria in abundance.

Taken together, our data provide evidence of substantial influences of smoking on the gut microbiota, which may in turn influence colorectal carcinogenesis.

In addition, we found that serum CEA and ALP of smoking-related enterotypes were significantly lower than those of non-smoking-related enterotypes. CEA is a well-known tumor marker associated with colorectal tumor progression. However, CEA may play a role as an innate immune defence that protects the colon from a broad load of microbes^35^. Serum ALP is partially derived from intestinal ALP. Intestinal ALP has innate immune functions such as detoxification of lipopolysaccharide, protection of intestinal barrier integrity, regulation of intestinal flora, and anti-inflammation^36^. Therefore, given the immunosuppressive effect of smoking^12,37,38^, this would explain why smoking has a stronger carcinogenic effect on smoking-associated enterotypes with lower CEA or lower ALP compared with non-smoking-associated enterotypes.

### Strengths and limitations

There are several strengths in this study. First, every individual enrolled in this study, including the healthy persons in the control group, underwent a complete colonoscopy with full visualization of the colon from cecum to rectum, and colonoscopy is regarded as the most robust reference standard for the presence or absence of polyps. Second, in addition to CRC cases, patients with adenoma, a critical precursor to CRC, were also included in this study. This provides us a chance to observe dynamic shifts in microbial composition and function during multistep CRC progression, and further confirm the effect of smoking on CRC development. Finally, our study included predictive functional analyses based on the microbial communities of the smoking-related and non-smoking-related subgroups. This effort suggested key insights into how the host and microbial community may interact within the context of CRC development.

There are some limitations in this study. First, the weaknesses of this study included those intrinsic to retrospective studies, including selection bias and recall bias. Second, the lack of replication is also a weakness. Further replications of similar case-control studies in different regions and populations or large-scale prospective cohort studies are needed to confirm our findings. Third, our study may be subject to differential exposure bias due to reverse causation. This means that patients with CRC or adenoma likely changed diets due to symptoms close to diagnosis, which changed the microbiome. Therefore, a prospective cohort study design that enables stool collection before colorectal neoplasm detection should be considered to address reverse causation bias^39^. Fourth, we only examined the fecal microbiota and not the mucosal-associated microbiota, which has been reported to differ in composition and diversity^40^. Fifth, we only conducted 16S rDNA sequencing and not metagenomic and metabolomic analyses, resulting in the inability to obtain a more accurate microbial community at the species level and real microbial functions. Finally, tumor microsatellite instability (MSI) status and immune cells, which also play an important role in the generation of intratumor heterogeneity, were not included in our analysis.

### Implications

Evidence indicates that smoking may increase the risk of MSI-high CRC^41^, which is characterized by an intense immune response to the tumor^42^. Recently a link between smoking and immune suppression has been well described^12,37,38^. The gut microbiota potentially influences tumor phenotypes directly or indirectly by modulating the host’s local and systemic antitumor immunity^39^. Since smoking, tumor MSI status, and gut microbiota affect tumor-immune interactions, further integrative analyses of those factors and immunity, called the integrated molecular pathological epidemiological (MPE) approach^43^, should be performed to confirm our findings. MPE research can be a promising direction^39,43,44^. By using the MPE approach, tumor subtype analyses based on specific microbial features, and molecular and pathological signatures can be further integrated into epidemiological studies to gain deeper ideas on CRC pathogenesis. The MPE approach enables us to test specific etiological hypotheses that connect exogenous or endogenous factors (such as smoking) to molecular pathology and a specific CRC subtype, thereby augmenting causal inference^44^. In addition, MPE research can be used to assess not only well-defined clinical outcomes (such as disease incidence and mortality) but also intermediary biomarkers that can predict full-blown disease in the future^44^.

## CONCLUSIONS

This study is the first to prove that smoking is associated with higher odds of type II colorectal neoplasms but not type I tumors classified by the gut microbiota. Our data also generate new evidence about how the gut microbiota may mediate the association of smoking with colorectal neoplasms. Further large-scale, prospective studies are needed to confirm these findings.

## Data Availability

The data supporting this research are available from the authors on reasonable request. Data supporting the findings of this study are also available within the article and the Supplementary file.
