# Supplementary material for: Association of cigarette smoking with risk of colorectal cancer subtypes classified by gut microbiota
Source: Tob Induc Dis. 2023 Aug 1;21:99. doi: 10.18332/tid/168515 (PMC10377954; doi:10.18332/tid/168515)
Supplement: Supplementary file 1 [file TID-21-99-s1.pdf]

Supplementary Materials

Supplementary Methods

To identify potential microbiota markers that differentiate between the two subtypes of colorectal cancer or colorectal adenoma, we constructed classification models based on the top 30 different genera using two different methods, linear support vector machine and logistic regression, respectively. All analyses were carried out using the Python package 'scikit-learn'[1].

The SelectFromModel method from scikit-learn was used for feature selection. The mean absolute coefficient value is set as a threshold parameter, and if the corresponding absolute coefficient values are below the mean absolute value, the features are considered unimportant and removed. Models were validated by 5-fold stratified cross-validation testing (we resampled dataset partitions 5 times). In each test, the accuracy of the model was examined using an area under the receiver operating characteristic curve.

Supplementary References

1. Pedregosa F, Varoquaux G, Gramfort A, et al. Scikit-learn: machine learning in Python. *JMLR* 2011; 12: 2825-2830.

Supplementary Table

Supplementary Table 1. Comparison of clinical characteristics between two subgroups in the colorectal cancer group

Variable	Type I (n = 77)	Type II (n = 53)	*P* value
Age, years			0.499
Mean (SD)	60.05 (9.98)	61.25 (9.69)	
Range	41-88	40-80	
Sex, No. (%)			0.212
Female	35 (45.5)	30 (56.6)	
Male	42 (54.5)	23 (43.4)	
Body mass index [a], kg/m^2			0.489
Mean (SD)	23.75 (3.16)	23.37 (3.00)	
Range	17.03-33.33	17.02-30.85	
Education degree, No. (%)			0.990
Illiteracy	9 (11.7)	6 (11.3)	
Primary	12 (15.6)	8 (15.1)	
Middle	46 (59.7)	33 (62.3)	
High	10 (13.0)	6 (11.3)	
Physical activity, No. (%)			0.228
Heavy	12 (15.6)	12 (22.6)	
Medium	17 (22.1)	14 (26.4)	
Light	48 (62.3)	27 (51.0)	

Diseases history, No. (%)			
Hypertension			0.380
No	55 (71.4)	34 (64.2)	
Yes	22 (28.6)	19 (35.8)	
Coronary heart disease			0.201
No	74 (96.1)	48 (90.6)	
Yes	3 (3.9)	5 (9.4)	
Diabetes			0.677
No	70 (90.9)	47 (88.7)	
Yes	7 (9.1)	6 (11.3)	
TNM stage, No. (%)			0.422
Stage I-II	47 (61.0)	36 (67.9)	
Stage III-IV	30 (39.0)	17 (32.1)	
Differentiation grade, No. (%)			0.058
Unevaluable	0 (0.0)	1 (1.9)	
Low	0 (0.0)	3 (5.7)	
Medium	76 (98.7)	48 (90.6)	
High	1 (1.3)	1 (1.9)	
Lesion site, No. (%)			0.689
Proximal colon [b]	25 (32.5)	19 (35.8)	
Distal colon [b] and rectum	52 (67.5)	34 (64.2)	

Complete blood count			
Lymphocyte count, $\times 10^9$/L			0.818
Mean (SD)	1.88 (0.74)	1.85 (0.62)	
Range	0.75-4.47	0.55-3.6	
Neutrophil count, $\times 10^9$/L			0.656
Mean (SD)	3.97 (1.5)	3.84 (1.92)	
Range	1.37-8.24	1.2-12.34	
Platelet count, $\times 10^9$/L			0.073
Mean (SD)	239.52 (72.01)	218.02 (57.77)	
Range	100-418	88-347	
Hemoglobin, g/L			0.402
Mean (SD)	119.65 (25.19)	122.79 (17.44)	
Range	53-177	77-161	
Blood biochemistry			
Direct bilirubin, μmol/L			0.235
Mean (SD)	3.19 (1.70)	4.72 (11.08)	
Range	0.5-9.7	1-83	
Albumin, g/L			0.580
Mean (SD)	39.57 (3.41)	39.25 (3.11)	
Range	32-49	30-47	
Alanine transaminase, U/L			0.506
Mean (SD)	19.86 (11.6)	21.92 (23.4)	
Range	5-75	6-173	

Aspartate aminotransferase, U/L			0.411
Mean (SD)	21.45 (6.48)	23.26 (17.62)	
Range	13-42	11-136	
Alkaline phosphatase, U/L			0.048
Mean (SD)	69.97 (19.14)	63.83 (15.28)	
Range	20-153	42-102	
Lactic dehydrogenase, U/L			0.135
Mean (SD)	170.99 (41.68)	162.15 (25.09)	
Range	102-324	86-229	
Fasting blood-glucose, mmol/L			0.278
Mean (SD)	5.28 (1.05)	6 (5.69)	
Range	4-9	4-46	
Creatinine, μmol/L			0.396
Mean (SD)	71.22 (14.19)	68.92 (16.34)	
Range	46-99	39-114	
Tumor markers			
Carcinoembryonic antigen, ng/ml			0.039
Mean (SD)	11.55 (23.83)	5.45 (7.19)	
Range	0.68-157.98	0.79-35.18	
Carbohydrate antigen 199, U/ml			0.719
Mean (SD)	58.41 (193.61)	46.9 (154.1)	
Range	2-999.99	2-922.02	

Abbreviations: SD, standard deviation.

[a] Body mass index: weight (kg)/height (m)^2.

[b] With the splenic flexure as the boundary, the proximal colon includes the cecum, ascending colon, hepatic flexure, transverse colon, and splenic flexure, while the distal colon includes the descending colon, sigmoid colon, and the rectosigmoid junction.

Supplementary Table 2. Comparison of clinical characteristics between two subgroups in the colorectal adenoma group

Variable	Type I (n = 66)	Type II (n = 54)	*P* value
Age, years			0.461
Mean (SD)	58.44 (9.59)	59.81 (10.75)	
Range	41-83	40-84	
Sex, No. (%)			0.723
Female	26 (39.4)	23 (42.6)	
Male	40 (60.6)	31 (57.4)	
Body mass index [a], kg/m^2			0.766
Mean (SD)	23.95 (3.28)	24.13 (3.42)	
Range	15.43-32.24	17.30-31.14	
Education degree, No. (%)			0.404
Illiteracy	2 (3.0)	3 (5.6)	
Primary	9 (13.6)	13 (24.1)	
Middle	39 (59.1)	26 (48.1)	
High	16 (24.2)	12 (22.2)	

Physical activity, No. (%)			0.966
Heavy	5 (7.6)	5 (9.3)	
Medium	13 (19.7)	12 (22.2)	
Light	48 (72.8)	37 (68.5)	
Diseases history, No. (%)			
Hypertension			0.889
No	46 (69.7)	37 (68.5)	
Yes	20 (30.3)	17 (31.5)	
Coronary heart disease			0.507
No	63 (95.5)	50 (92.6)	
Yes	3 (4.5)	4 (7.4)	
Diabetes			0.459
No	60 (90.9)	51 (94.4)	
Yes	6 (9.1)	3 (5.6)	
Lesion site, No. (%)			0.729
Proximal colon [b]	24 (36.4)	18 (33.3)	
Distal colon [b] and rectum	42 (63.6)	36 (66.7)	
Complete blood count			
Lymphocyte count, $\times 10^9$/L			0.721
Mean (SD)	2.13 (0.74)	2.18 (0.72)	
Range	0.55-4.57	0.87-3.98	

Neutrophil count, ×10^9/L			0.343
Mean (SD)	3.74 (1.22)	3.97 (1.48)	
Range	1.21-6.02	1.56-8.44	
Platelet count, ×10^9/L			0.250
Mean (SD)	195.95 (50.94)	206.57 (48.86)	
Range	89-303	124-380	
Hemoglobin, g/L			0.734
Mean (SD)	140.11 (17.80)	139.19 (11.57)	
Range	71-172	115-157	
Blood biochemistry			
Direct bilirubin, μmol/L			0.744
Mean (SD)	3.62 (1.85)	3.44 (1.35)	
Range	1.2-9.1	1.7-7.3	
Albumin, g/L			0.642
Mean (SD)	40.08 (2.96)	39.08 (9.88)	
Range	34-45	4-49	
Alanine transaminase, U/L			0.160
Mean (SD)	27.17 (21.17)	19.34 (6.17)	
Range	12-121	8-28	
Aspartate aminotransferase, U/L			0.242
Mean (SD)	27.79 (21.44)	21.17 (7.13)	
Range	15-126	13-43	

Alkaline phosphatase, U/L			0.039
Mean (SD)	71.21 (24.61)	56.54 (14.65)	
Range	25-143	29-75	
Lactic dehydrogenase, U/L			0.265
Mean (SD)	167.08 (22.36)	175.4 (22.25)	
Range	133-220	145-241	
Fasting blood-glucose, mmol/L			0.695
Mean (SD)	5.01 (0.74)	4.88 (1.37)	
Range	4-7	2-9	
Creatinine, μmol/L			0.277
Mean (SD)	78.78 (16.33)	73.11 (14.99)	
Range	43-111	51-97	
Tumor markers			
Carcinoembryonic antigen, ng/ml			0.008
Mean (SD)	3.55 (2.39)	2 (0.77)	
Range	1.03-9.28	0.88-3.81	
Carbohydrate antigen 199, U/ml			0.192
Mean (SD)	12.17 (13.11)	8.12 (4.7)	
Range	2-55.09	2-21.36	

Abbreviations: SD, standard deviation.

[a] Body mass index: weight (kg)/height (m)^2.

[b] With the splenic flexure as the boundary, the proximal colon includes the cecum, ascending colon, hepatic flexure, transverse colon, and splenic flexure, while the distal colon includes the descending colon, sigmoid colon, and the rectosigmoid junction.

Supplementary Table 3. Correlations between differential genera in type I colorectal cancer (Type I VS. Type II)

Genus 1	Genus 2	*P* value	Correlation coefficient
Adlercreutzia	*Christensenellaceae R-7 group*	0.028	0.251
Adlercreutzia	*Eubacterium coprostanoligenes group*	0.038	0.237
Adlercreutzia	*Oxalobacter*	0.001	0.361
Eggerthella	*Eubacterium ruminantium group*	0.001	-0.384
Eggerthella	*Coprococcus 2*	0.026	-0.254
Eggerthella	*Lachnoclostridium*	0.001	0.367
Eggerthella	*Lachnospiraceae UCG-001*	0.002	-0.345
Eggerthella	*Lachnospiraceae UCG-005*	0.046	-0.228
Eggerthella	*Marvinbryantia*	0.039	-0.236
Eggerthella	*Eubacterium coprostanoligenes group*	0.001	-0.362
Eggerthella	*Ruminiclostridium 6*	<0.001	-0.448
Eggerthella	*Ruminococcaceae UCG-002*	<0.001	-0.392
Eggerthella	*Ruminococcaceae UCG-009*	0.007	-0.305
Eggerthella	*Ruminococcaceae UCG-010*	<0.001	-0.503
Eggerthella	*Ruminococcaceae UCG-014*	<0.001	-0.412
Eggerthella	*Ruminococcus 1*	0.035	-0.241
Eggerthella	*Erysipelotrichaceae Incertae Sedis*	<0.001	0.474
Eggerthella	*Victivallis*	0.001	-0.362

Eggerthella	*Oxalobacter*	0.011	-0.289
Porphyromonas	*Lachnospiraceae NK4A136 group*	0.015	-0.276
Porphyromonas	*Hydrogenoanaerobacterium*	0.039	0.236
Prevotella 2	*Lachnospiraceae NK4A136 group*	0.022	-0.260
Prevotella 2	*Lachnospiraceae UCG-001*	0.021	-0.262
Prevotella 2	*Ruminiclostridium 9*	0.025	0.256
Prevotella 2	*Ruminococcus 1*	0.008	-0.299
Christensenellaceae R-7 group	*Eubacterium ruminantium group*	0.004	0.326
Christensenellaceae R-7 group	*Coprococcus 2*	<0.001	0.485
Christensenellaceae R-7 group	*Lachnoclostridium*	0.002	-0.340
Christensenellaceae R-7 group	*Lachnospiraceae UCG-001*	0.001	0.374
Christensenellaceae R-7 group	*Lachnospiraceae UCG-005*	0.048	0.226
Christensenellaceae R-7 group	*Marvinbryantia*	<0.001	0.405
Christensenellaceae R-7 group	*Peptococcus*	0.001	0.387
Christensenellaceae R-7 group	*Eubacterium coprostanoligenes group*	<0.001	0.412
Christensenellaceae R-7 group	*Hydrogenoanaerobacterium*	<0.001	0.586
Christensenellaceae R-7 group	*Ruminiclostridium 6*	<0.001	0.444
Christensenellaceae R-7 group	*Ruminococcaceae NK4A214 group*	<0.001	0.497
Christensenellaceae R-7 group	*Ruminococcaceae UCG-002*	<0.001	0.521
Christensenellaceae R-7 group	*Ruminococcaceae UCG-009*	0.003	0.336
Christensenellaceae R-7 group	*Ruminococcaceae UCG-010*	<0.001	0.653

Christensenellaceae R-7 group	*Ruminococcaceae UCG-014*	<0.001	0.573
Christensenellaceae R-7 group	*Ruminococcus 1*	0.002	0.342
Christensenellaceae R-7 group	*Erysipelotrichaceae Incertae Sedis*	0.030	-0.248
Christensenellaceae R-7 group	*Victivallis*	<0.001	0.445
Christensenellaceae R-7 group	*Oxalobacter*	0.001	0.375
Christensenellaceae R-7 group	*Cloacibacillus*	0.029	0.249
Eubacterium ruminantium group	*Coprococcus 2*	<0.001	0.420
Eubacterium ruminantium group	*Lachnoclostridium*	0.002	-0.344
Eubacterium ruminantium group	*Lachnospiraceae NK4A136 group*	0.034	0.242
Eubacterium ruminantium group	*Lachnospiraceae UCG-001*	<0.001	0.430
Eubacterium ruminantium group	*Marvinbryantia*	0.022	0.261
Eubacterium ruminantium group	*Eubacterium coprostanoligenes group*	0.017	0.272
Eubacterium ruminantium group	*Ruminiclostridium 6*	<0.001	0.526
Eubacterium ruminantium group	*Ruminococcaceae UCG-002*	0.026	0.254
Eubacterium ruminantium group	*Ruminococcaceae UCG-009*	0.001	0.373
Eubacterium ruminantium group	*Ruminococcaceae UCG-010*	0.003	0.332
Eubacterium ruminantium group	*Ruminococcaceae UCG-014*	<0.001	0.433
Eubacterium ruminantium group	*Ruminococcus 1*	0.003	0.334
Eubacterium ruminantium group	*Erysipelotrichaceae Incertae Sedis*	<0.001	-0.401
Eubacterium ruminantium group	*Victivallis*	0.031	0.247
Eubacterium ruminantium group	*Escherichia Shigella*	0.043	-0.231

Eubacterium ventriosum group	*Lachnoclostridium*	0.025	-0.255
Eubacterium ventriosum group	*Lachnospiraceae UCG-001*	0.030	0.248
Eubacterium ventriosum group	*Lachnospiraceae UCG-005*	0.002	0.345
Eubacterium ventriosum group	*Ruminiclostridium 6*	0.045	0.229
Eubacterium ventriosum group	*Ruminiclostridium 9*	0.001	-0.373
Eubacterium ventriosum group	*Ruminococcaceae NK4A214 group*	0.049	0.225
Eubacterium ventriosum group	*Ruminococcus 1*	0.011	0.289
Coprococcus 2	*Lachnoclostridium*	0.004	-0.322
Coprococcus 2	*Lachnospiraceae NK4A136 group*	0.022	0.262
Coprococcus 2	*Lachnospiraceae UCG-001*	<0.001	0.528
Coprococcus 2	*Lachnospiraceae UCG-005*	0.044	0.230
Coprococcus 2	*Marvinbryantia*	0.031	0.246
Coprococcus 2	*Peptococcus*	0.027	0.251
Coprococcus 2	*Eubacterium coprostanoligenes group*	0.013	0.283
Coprococcus 2	*Hydrogenoanaerobacterium*	0.002	0.346
Coprococcus 2	*Ruminiclostridium 6*	0.002	0.351
Coprococcus 2	*Ruminococcaceae NK4A214 group*	0.002	0.352
Coprococcus 2	*Ruminococcaceae UCG-002*	0.010	0.292
Coprococcus 2	*Ruminococcaceae UCG-009*	0.025	0.256
Coprococcus 2	*Ruminococcaceae UCG-010*	<0.001	0.527
Coprococcus 2	*Ruminococcaceae UCG-014*	<0.001	0.537

Coprococcus 2	*Ruminococcus 1*	<0.001	0.411
Coprococcus 2	*Erysipelotrichaceae Incertae Sedis*	0.001	-0.386
Coprococcus 2	*Escherichia Shigella*	0.026	-0.253
Lachnoclostridium	*Lachnospiraceae UCG-001*	0.014	-0.279
Lachnoclostridium	*Eubacterium coprostanoligenes group*	0.004	-0.322
Lachnoclostridium	*Hydrogenoanaerobacterium*	0.029	-0.250
Lachnoclostridium	*Ruminiclostridium 6*	0.026	-0.253
Lachnoclostridium	*Ruminococcaceae NK4A214 group*	<0.001	-0.416
Lachnoclostridium	*Ruminococcaceae UCG-002*	0.003	-0.336
Lachnoclostridium	*Ruminococcaceae UCG-009*	0.002	-0.356
Lachnoclostridium	*Ruminococcaceae UCG-010*	<0.001	-0.393
Lachnoclostridium	*Ruminococcaceae UCG-014*	0.014	-0.279
Lachnoclostridium	*Ruminococcus 1*	0.001	-0.358
Lachnoclostridium	*Erysipelotrichaceae Incertae Sedis*	<0.001	0.589
Lachnoclostridium	*Victivallis*	0.007	-0.306
Lachnoclostridium	*Escherichia Shigella*	<0.001	0.388
Lachnospiraceae NK4A136 group	*Lachnospiraceae UCG-001*	0.001	0.373
Lachnospiraceae NK4A136 group	*Ruminiclostridium 9*	0.001	-0.357
Lachnospiraceae NK4A136 group	*Ruminococcaceae UCG-014*	0.027	0.252
Lachnospiraceae NK4A136 group	*Ruminococcus 1*	0.023	0.259
Lachnospiraceae NK4A136 group	*Erysipelotrichaceae Incertae Sedis*	0.011	-0.290

Lachnospiraceae NK4A136 group	*Escherichia Shigella*	0.033	-0.243
Lachnospiraceae UCG-001	*Lachnospiraceae UCG-005*	0.001	0.368
Lachnospiraceae UCG-001	*Peptococcus*	0.007	0.303
Lachnospiraceae UCG-001	*Eubacterium coprostanoligenes group*	0.012	0.286
Lachnospiraceae UCG-001	*Ruminiclostridium 6*	0.003	0.334
Lachnospiraceae UCG-001	*Ruminiclostridium 9*	0.003	-0.330
Lachnospiraceae UCG-001	*Ruminococcaceae NK4A214 group*	0.001	0.382
Lachnospiraceae UCG-001	*Ruminococcaceae UCG-002*	<0.001	0.394
Lachnospiraceae UCG-001	*Ruminococcaceae UCG-010*	<0.002	0.432
Lachnospiraceae UCG-001	*Ruminococcaceae UCG-014*	<0.003	0.403
Lachnospiraceae UCG-001	*Ruminococcus 1*	<0.001	0.479
Lachnospiraceae UCG-001	*Erysipelotrichaceae Incertae Sedis*	0.002	-0.351
Lachnospiraceae UCG-001	*Oxalobacter*	0.035	0.240
Lachnospiraceae UCG-005	*Marvinbryantia*	0.012	0.286
Lachnospiraceae UCG-005	*Ruminococcaceae UCG-002*	0.028	0.251
Lachnospiraceae UCG-005	*Ruminococcaceae UCG-014*	0.002	0.349
Marvinbryantia	*Hydrogenoanaerobacterium*	0.016	0.273
Marvinbryantia	*Ruminiclostridium 6*	0.034	0.242
Marvinbryantia	*Ruminococcaceae NK4A214 group*	0.015	0.275
Marvinbryantia	*Ruminococcaceae UCG-002*	<0.001	0.457
Marvinbryantia	*Ruminococcaceae UCG-009*	0.003	0.337

Marvinbryantia	*Ruminococcaceae UCG-010*	0.002	0.351
Marvinbryantia	*Ruminococcaceae UCG-014*	0.002	0.348
Marvinbryantia	*Ruminococcus 1*	0.018	0.269
Marvinbryantia	*Victivallis*	0.002	0.341
Peptococcus	*Hydrogenoanaerobacterium*	<0.001	0.439
Peptococcus	*Ruminococcaceae UCG-002*	0.008	0.299
Peptococcus	*Ruminococcaceae UCG-010*	0.001	0.384
Peptococcus	*Ruminococcaceae UCG-014*	0.011	0.290
Peptococcus	*Erysipelotrichaceae Incertae Sedis*	0.016	-0.275
Eubacterium coprostanoligenes group	*Hydrogenoanaerobacterium*	0.010	0.292
Eubacterium coprostanoligenes group	*Ruminiclostridium 6*	<0.001	0.426
Eubacterium coprostanoligenes group	*Ruminococcaceae NK4A214 group*	<0.001	0.540
Eubacterium coprostanoligenes group	*Ruminococcaceae UCG-002*	<0.001	0.440
Eubacterium coprostanoligenes group	*Ruminococcaceae UCG-009*	0.001	0.380
Eubacterium coprostanoligenes group	*Ruminococcaceae UCG-010*	<0.001	0.396
Eubacterium coprostanoligenes group	*Ruminococcaceae UCG-014*	0.048	0.226
Eubacterium coprostanoligenes group	*Ruminococcus 1*	0.001	0.364
Eubacterium coprostanoligenes group	*Erysipelotrichaceae Incertae Sedis*	0.011	-0.288
Eubacterium coprostanoligenes group	*Victivallis*	0.007	0.306
Hydrogenoanaerobacterium	*Ruminiclostridium 6*	0.002	0.355
Hydrogenoanaerobacterium	*Ruminococcaceae NK4A214 group*	<0.001	0.476

Hydrogenoanaerobacterium	*Ruminococcaceae UCG-002*	<0.001	0.438
Hydrogenoanaerobacterium	*Ruminococcaceae UCG-010*	<0.001	0.453
Hydrogenoanaerobacterium	*Ruminococcaceae UCG-014*	<0.001	0.420
Hydrogenoanaerobacterium	*Victivallis*	0.012	0.284
Hydrogenoanaerobacterium	*Oxalobacter*	0.004	0.324
Hydrogenoanaerobacterium	*Cloacibacillus*	0.011	0.290
Ruminiclostridium 6	*Ruminococcaceae NK4A214 group*	0.038	0.237
Ruminiclostridium 6	*Ruminococcaceae UCG-002*	0.009	0.296
Ruminiclostridium 6	*Ruminococcaceae UCG-009*	0.001	0.359
Ruminiclostridium 6	*Ruminococcaceae UCG-010*	<0.001	0.522
Ruminiclostridium 6	*Ruminococcaceae UCG-014*	<0.001	0.420
Ruminiclostridium 6	*Ruminococcus 1*	0.002	0.355
Ruminiclostridium 6	*Erysipelotrichaceae Incertae Sedis*	0.027	-0.252
Ruminiclostridium 6	*Victivallis*	<0.001	0.398
Ruminiclostridium 6	*Pyramidobacter*	0.035	0.240
Ruminococcaceae NK4A214 group	*Ruminococcaceae UCG-002*	<0.001	0.582
Ruminococcaceae NK4A214 group	*Ruminococcaceae UCG-009*	<0.001	0.424
Ruminococcaceae NK4A214 group	*Ruminococcaceae UCG-010*	<0.001	0.413
Ruminococcaceae NK4A214 group	*Ruminococcaceae UCG-014*	0.001	0.366
Ruminococcaceae NK4A214 group	*Ruminococcus 1*	<0.001	0.520
Ruminococcaceae NK4A214 group	*Erysipelotrichaceae Incertae Sedis*	0.003	-0.333

Ruminococcaceae NK4A214 group	*Victivallis*	0.018	0.270
Ruminococcaceae NK4A214 group	*Oxalobacter*	0.009	0.294
Ruminococcaceae UCG-002	*Ruminococcaceae UCG-009*	<0.001	0.475
Ruminococcaceae UCG-002	*Ruminococcaceae UCG-010*	<0.001	0.531
Ruminococcaceae UCG-002	*Ruminococcaceae UCG-014*	0.001	0.370
Ruminococcaceae UCG-002	*Ruminococcus 1*	0.001	0.356
Ruminococcaceae UCG-002	*Erysipelotrichaceae Incertae Sedis*	<0.001	-0.491
Ruminococcaceae UCG-002	*Victivallis*	0.001	0.372
Ruminococcaceae UCG-002	*Oxalobacter*	<0.001	0.416
Ruminococcaceae UCG-009	*Ruminococcaceae UCG-010*	<0.001	0.456
Ruminococcaceae UCG-009	*Ruminococcaceae UCG-014*	0.012	0.285
Ruminococcaceae UCG-009	*Ruminococcus 1*	0.020	0.265
Ruminococcaceae UCG-009	*Erysipelotrichaceae Incertae Sedis*	0.008	-0.302
Ruminococcaceae UCG-009	*Victivallis*	0.003	0.332
Ruminococcaceae UCG-009	*Escherichia Shigella*	0.014	-0.278
Ruminococcaceae UCG-010	*Ruminococcaceae UCG-014*	<0.001	0.559
Ruminococcaceae UCG-010	*Ruminococcus 1*	0.001	0.359
Ruminococcaceae UCG-010	*Erysipelotrichaceae Incertae Sedis*	<0.001	-0.408
Ruminococcaceae UCG-010	*Victivallis*	<0.001	0.550
Ruminococcaceae UCG-010	*Oxalobacter*	<0.001	0.447
Ruminococcaceae UCG-014	*Ruminococcus 1*	0.009	0.296

Ruminococcaceae UCG-014	*Erysipelotrichaceae Incertae Sedis*	0.005	-0.318
Ruminococcaceae UCG-014	*Victivallis*	<0.001	0.397
Ruminococcaceae UCG-014	*Oxalobacter*	0.032	0.244
Ruminococcus 1	*Erysipelotrichaceae Incertae Sedis*	0.004	-0.323
Ruminococcus 1	*Victivallis*	0.011	0.287
Ruminococcus 1	*Pyramidobacter*	0.029	0.250
Erysipelotrichaceae Incertae Sedis	*Escherichia Shigella*	<0.001	0.400
Victivallis	*Oxalobacter*	0.002	0.346
Oxalobacter	*Cloacibacillus*	<0.001	0.495

Supplementary Table 4. Correlations between differential genera in type II colorectal cancer (Type II VS. Type I)

Genus 1	Genus 2	*P* value	Correlation coefficient
Adlercreutzia	*Ruminiclostridium 6*	0.020	0.318
Eggerthella	*Lachnoclostridium*	<0.001	0.566
Eggerthella	*Ruminiclostridium 6*	0.005	-0.377
Eggerthella	*Ruminococcaceae UCG-014*	0.045	-0.276
Eggerthella	*Erysipelotrichaceae Incertae Sedis*	<0.001	0.494
Eggerthella	*Escherichia Shigella*	0.032	-0.296
Eggerthella	*Pyramidobacter*	0.016	0.331
Porphyromonas	*Prevotella 2*	0.014	0.336
Porphyromonas	*Christensenellaceae R-7 group*	0.036	0.289
Porphyromonas	*Ruminiclostridium 6*	0.001	0.439
Porphyromonas	*Ruminococcaceae NK4A214 group*	0.024	0.310
Porphyromonas	*Ruminococcaceae UCG-002*	0.001	0.442
Porphyromonas	*Ruminococcaceae UCG-010*	0.044	0.278
Porphyromonas	*Ruminococcaceae UCG-014*	0.008	0.359
Porphyromonas	*Victivallis*	0.035	0.290
Porphyromonas	*Escherichia Shigella*	0.019	0.321
Prevotella 2	*Eubacterium ruminantium group*	0.031	0.296
Prevotella 2	*Ruminiclostridium 6*	0.039	0.285

Prevotella 2	*Ruminococcaceae UCG-014*	0.001	0.442
Prevotella 2	*Oxalobacter*	0.004	0.385
Prevotella 2	*Cloacibacillus*	0.044	0.278
Christensenellaceae R-7 group	*Lachnospiraceae NK4A136 group*	0.015	0.332
Christensenellaceae R-7 group	*Marvinbryantia*	0.047	0.274
Christensenellaceae R-7 group	*Eubacterium coprostanoligenes group*	0.032	0.296
Christensenellaceae R-7 group	*Ruminiclostridium 9*	0.016	0.331
Christensenellaceae R-7 group	*Ruminococcaceae NK4A214 group*	0.007	0.369
Christensenellaceae R-7 group	*Ruminococcaceae UCG-002*	0.009	0.354
Christensenellaceae R-7 group	*Cloacibacillus*	0.008	0.359
Eubacterium ruminantium group	*Coprococcus 2*	<0.001	0.525
Eubacterium ruminantium group	*Ruminococcaceae UCG-014*	<0.001	0.578
Eubacterium ventriosum group	*Lachnospiraceae NK4A136 group*	<0.001	0.479
Eubacterium ventriosum group	*Lachnospiraceae UCG-001*	0.006	0.370
Eubacterium ventriosum group	*Lachnospiraceae UCG-005*	0.034	0.292
Eubacterium ventriosum group	*Marvinbryantia*	0.005	0.378
Eubacterium ventriosum group	*Eubacterium coprostanoligenes group*	0.001	0.438
Eubacterium ventriosum group	*Ruminiclostridium 9*	0.024	0.310
Eubacterium ventriosum group	*Cloacibacillus*	0.006	0.371
Coprococcus 2	*Ruminococcaceae UCG-014*	0.016	0.328
Lachnoclostridium	*Erysipelotrichaceae Incertae Sedis*	<0.001	0.480

Lachnospiraceae NK4A136 group	*Marvinbryantia*	0.025	0.309
Lachnospiraceae NK4A136 group	*Eubacterium coprostanoligenes group*	0.002	0.424
Lachnospiraceae UCG-001	*Eubacterium coprostanoligenes group*	0.019	0.322
Lachnospiraceae UCG-001	*Cloacibacillus*	0.018	0.323
Lachnospiraceae UCG-005	*Marvinbryantia*	0.007	0.368
Marvinbryantia	*Ruminiclostridium 9*	0.032	0.295
Eubacterium coprostanoligenes group	*Ruminococcaceae NK4A214 group*	0.026	0.307
Eubacterium coprostanoligenes group	*Ruminococcaceae UCG-002*	0.003	0.407
Eubacterium coprostanoligenes group	*Ruminococcaceae UCG-009*	0.002	0.407
Eubacterium coprostanoligenes group	*Ruminococcaceae UCG-010*	0.042	0.280
Hydrogenoanaerobacterium	*Ruminococcaceae NK4A214 group*	0.004	0.388
Hydrogenoanaerobacterium	*Ruminococcaceae UCG-009*	0.002	0.408
Hydrogenoanaerobacterium	*Oxalobacter*	0.042	0.280
Ruminiclostridium 6	*Ruminococcaceae UCG-014*	<0.001	0.565
Ruminiclostridium 6	*Ruminococcus 1*	0.006	0.370
Ruminiclostridium 6	*Escherichia Shigella*	0.001	0.429
Ruminiclostridium 9	*Ruminococcaceae UCG-009*	0.017	0.327
Ruminococcaceae NK4A214 group	*Ruminococcaceae UCG-002*	0.001	0.445
Ruminococcaceae NK4A214 group	*Ruminococcaceae UCG-010*	0.042	0.281
Ruminococcaceae NK4A214 group	*Victivallis*	0.031	0.297
Ruminococcaceae NK4A214 group	*Oxalobacter*	0.014	0.335

Ruminococcaceae UCG-002	*Ruminococcaceae UCG-010*	0.014	0.337
Ruminococcaceae UCG-002	*Ruminococcus 1*	0.026	0.305
Ruminococcaceae UCG-009	*Victivallis*	0.001	0.429
Ruminococcaceae UCG-010	*Victivallis*	<0.001	0.588
Ruminococcaceae UCG-014	*Ruminococcus 1*	0.023	0.312
Ruminococcaceae UCG-014	*Escherichia Shigella*	<0.001	0.502
Ruminococcus 1	*Victivallis*	0.045	0.276
Erysipelotrichaceae Incertae Sedis	*Pyramidobacter*	0.010	0.352

Supplementary Table 5. Correlations between differential genera in type I colorectal adenoma (Type I VS. Type II)

Genus 1	Genus 2	*P* value	Correlation coefficient
Actinomyces	*Lactococcus*	0.001	0.394
Actinomyces	*Lachnoclostridium*	0.002	0.369
Actinomyces	*Erysipelatoclostridium*	0.001	0.394
Actinomyces	*Erysipelotrichaceae Incertae Sedis*	0.031	0.265
Adlercreutzia	*Coprobacter*	0.021	0.283
Adlercreutzia	*Eubacterium brachy group*	<0.001	0.435
Adlercreutzia	*Lactonifactor*	0.005	0.345
Adlercreutzia	*Ruminiclostridium 6*	0.044	0.249
Adlercreutzia	*Ruminococcaceae NK4A214 group*	0.004	0.351
Adlercreutzia	*Fusobacterium*	0.017	-0.294
Adlercreutzia	*Oxalobacter*	0.019	0.289
Coprobacter	*Eubacterium brachy group*	0.010	0.315
Coprobacter	*Lachnoclostridium*	0.009	0.320
Bacillus	*Lactococcus*	0.001	0.398
Bacillus	*Escherichia Shigella*	0.010	0.316
Bacillus	*Candidatus Saccharimonas*	0.035	0.259
Enterococcus	*Escherichia Shigella*	<0.001	0.423
Lactococcus	*Lachnoclostridium*	0.039	0.255
Lactococcus	*Erysipelotrichaceae Incertae Sedis*	0.024	0.278

Lactococcus	*Escherichia Shigella*	0.020	0.286
Lactococcus	*Candidatus Saccharimonas*	0.040	0.253
Christensenellaceae R-7 group	*Eubacterium brachy group*	0.009	0.319
Christensenellaceae R-7 group	*Family XIII UCG-001*	0.011	0.313
Christensenellaceae R-7 group	*Coprococcus 2*	<0.001	0.480
Christensenellaceae R-7 group	*Lachnoclostridium*	0.028	-0.270
Christensenellaceae R-7 group	*Ruminiclostridium 6*	0.001	0.395
Christensenellaceae R-7 group	*Ruminococcaceae NK4A214 group*	<0.001	0.485
Christensenellaceae R-7 group	*Ruminococcaceae UCG-002*	<0.001	0.605
Christensenellaceae R-7 group	*Ruminococcaceae UCG-005*	<0.001	0.739
Christensenellaceae R-7 group	*Ruminococcaceae UCG-010*	<0.001	0.665
Christensenellaceae R-7 group	*Ruminococcaceae UCG-014*	<0.001	0.541
Christensenellaceae R-7 group	*Oxalobacter*	0.001	0.387
Eubacterium brachy group	*Lactonifactor*	<0.001	0.551
Eubacterium brachy group	*Ruminococcaceae UCG-005*	0.017	0.294
Eubacterium brachy group	*Ruminococcaceae UCG-010*	0.027	0.273
Eubacterium brachy group	*Oxalobacter*	<0.001	0.450
Family XIII UCG-001	*Ruminococcaceae NK4A214 group*	0.002	0.380
Family XIII UCG-001	*Ruminococcaceae UCG-002*	0.020	0.286
Family XIII UCG-001	*Ruminococcaceae UCG-005*	<0.001	0.435
Family XIII UCG-001	*Ruminococcaceae UCG-010*	0.015	0.298

Family XIII UCG-001	*Erysipelotrichaceae Incertae Sedis*	0.022	-0.282
Family XIII UCG-001	*Fusobacterium*	0.030	-0.268
Coprococcus 2	*Ruminiclostridium 6*	0.001	0.399
Coprococcus 2	*Ruminococcaceae NK4A214 group*	0.002	0.381
Coprococcus 2	*Ruminococcaceae UCG-002*	0.033	0.263
Coprococcus 2	*Ruminococcaceae UCG-005*	0.002	0.369
Coprococcus 2	*Ruminococcaceae UCG-010*	<0.001	0.527
Coprococcus 2	*Ruminococcaceae UCG-014*	0.002	0.373
Coprococcus 2	*Fusobacterium*	0.028	-0.270
Coprococcus 2	*Oxalobacter*	0.027	0.271
Lachnoclostridium	*Ruminiclostridium 6*	0.049	-0.244
Lachnoclostridium	*Ruminococcaceae NK4A214 group*	0.038	-0.257
Lachnoclostridium	*Ruminococcaceae UCG-014*	0.023	-0.280
Lachnoclostridium	*Erysipelatoclostridium*	0.008	0.322
Lachnoclostridium	*Erysipelotrichaceae Incertae Sedis*	0.025	0.275
Lactonifactor	*Ruminococcaceae UCG-005*	0.038	0.256
Lactonifactor	*Erysipelatoclostridium*	0.009	0.318
Ruminiclostridium 6	*Ruminococcaceae NK4A214 group*	0.024	0.277
Ruminiclostridium 6	*Ruminococcaceae UCG-005*	0.004	0.350
Ruminiclostridium 6	*Ruminococcaceae UCG-010*	<0.001	0.454
Ruminiclostridium 6	*Ruminococcaceae UCG-014*	0.010	0.313

Ruminiclostridium 6	*Escherichia Shigella*	0.021	0.283
Ruminococcaceae NK4A214 group	*Ruminococcaceae UCG-002*	0.001	0.414
Ruminococcaceae NK4A214 group	*Ruminococcaceae UCG-005*	<0.001	0.541
Ruminococcaceae NK4A214 group	*Ruminococcaceae UCG-010*	<0.001	0.420
Ruminococcaceae NK4A214 group	*Ruminococcaceae UCG-014*	0.022	0.282
Ruminococcaceae NK4A214 group	*Megamonas*	0.020	-0.286
Ruminococcaceae NK4A214 group	*Fusobacterium*	0.033	-0.263
Ruminococcaceae NK4A214 group	*Oxalobacter*	0.012	0.308
Ruminococcaceae UCG-002	*Ruminococcaceae UCG-005*	<0.001	0.668
Ruminococcaceae UCG-002	*Ruminococcaceae UCG-010*	0.008	0.322
Ruminococcaceae UCG-002	*Ruminococcaceae UCG-014*	0.017	0.292
Ruminococcaceae UCG-002	*Oxalobacter*	0.045	0.248
Ruminococcaceae UCG-005	*Ruminococcaceae UCG-010*	<0.001	0.542
Ruminococcaceae UCG-005	*Ruminococcaceae UCG-014*	<0.001	0.485
Ruminococcaceae UCG-005	*Oxalobacter*	0.015	0.299
Ruminococcaceae UCG-010	*Ruminococcaceae UCG-014*	<0.001	0.494
Ruminococcaceae UCG-010	*Thalassospira*	0.047	0.246
Ruminococcaceae UCG-010	*Oxalobacter*	<0.001	0.437
Ruminococcaceae UCG-014	*Thalassospira*	0.041	0.253
Erysipelatoclostridium	*Erysipelotrichaceae Incertae Sedis*	<0.001	0.470
Megamonas	*Fusobacterium*	<0.001	0.427

Supplementary Table 6. Correlations between differential genera in type II colorectal adenoma (Type II VS. Type I)

Genus 1	Genus 2	*P* value	Correlation coefficient
Actinomyces	*Bacillus*	0.004	0.385
Actinomyces	*Lactococcus*	0.011	0.345
Actinomyces	*Lachnoclostridium*	0.004	0.388
Actinomyces	*Oribacterium*	<0.001	0.612
Actinomyces	*Erysipelatoclostridium*	0.027	0.300
Actinomyces	*Candidatus Saccharimonas*	0.001	0.439
Adlercreutzia	*Ruminococcaceae NK4A214 group*	0.006	0.368
Adlercreutzia	*Ruminococcaceae UCG-002*	0.026	0.302
Adlercreutzia	*Ruminococcaceae UCG-010*	0.001	0.441
Adlercreutzia	*Ruminococcaceae UCG-014*	0.025	0.304
Adlercreutzia	*Thalassospira*	0.006	0.372
Coprobacter	*Erysipelotrichaceae Incertae Sedis*	0.037	0.284
Bacillus	*Lactococcus*	<0.001	0.660
Bacillus	*Lachnoclostridium*	<0.001	0.552
Bacillus	*Stomatobaculum*	0.023	0.310
Bacillus	*Erysipelotrichaceae Incertae Sedis*	0.039	0.282
Bacillus	*Escherichia Shigella*	0.003	0.392
Bacillus	*Candidatus Saccharimonas*	0.001	0.452
Enterococcus	*Erysipelotrichaceae Incertae Sedis*	0.007	0.362

Enterococcus	*Escherichia Shigella*	0.022	0.311
Lactococcus	*Oribacterium*	0.009	0.351
Lactococcus	*Stomatobaculum*	0.008	0.357
Lactococcus	*Escherichia Shigella*	0.007	0.365
Lactococcus	*Candidatus Saccharimonas*	0.034	0.289
Christensenellaceae R-7 group	*Coprococcus 2*	0.001	0.453
Christensenellaceae R-7 group	*Stomatobaculum*	0.002	0.406
Christensenellaceae R-7 group	*Ruminiclostridium 6*	<0.001	0.484
Christensenellaceae R-7 group	*Ruminococcaceae NK4A214 group*	<0.001	0.482
Christensenellaceae R-7 group	*Ruminococcaceae UCG-002*	<0.001	0.546
Christensenellaceae R-7 group	*Ruminococcaceae UCG-005*	<0.001	0.626
Christensenellaceae R-7 group	*Ruminococcaceae UCG-010*	0.001	0.429
Christensenellaceae R-7 group	*Ruminococcaceae UCG-014*	0.041	0.279
Christensenellaceae R-7 group	*Thalassospira*	0.030	0.296
Christensenellaceae R-7 group	*Candidatus Saccharimonas*	0.024	0.306
Eubacterium brachy group	*Oribacterium*	0.010	0.349
Eubacterium brachy group	*Stomatobaculum*	0.002	0.418
Eubacterium brachy group	*Erysipelatoclostridium*	0.034	0.290
Eubacterium brachy group	*Megamonas*	0.043	-0.277
Family XIII UCG-001	*Ruminococcaceae NK4A214 group*	0.009	0.355
Family XIII UCG-001	*Ruminococcaceae UCG-002*	0.045	0.274

Family XIII UCG-001	*Ruminococcaceae UCG-005*	<0.001	0.519
Coprococcus 2	*Ruminiclostridium 6*	<0.001	0.611
Coprococcus 2	*Ruminococcaceae NK4A214 group*	0.010	0.346
Coprococcus 2	*Ruminococcaceae UCG-010*	0.029	0.298
Coprococcus 2	*Candidatus Saccharimonas*	0.029	0.297
Lachnoclostridium	*Oribacterium*	0.034	0.289
Lachnoclostridium	*Erysipelatoclostridium*	0.017	0.322
Lachnoclostridium	*Erysipelotrichaceae Incertae Sedis*	0.002	0.411
Lachnoclostridium	*Fusobacterium*	0.037	0.284
Lachnoclostridium	*Escherichia Shigella*	<0.001	0.476
Lachnoclostridium	*Candidatus Saccharimonas*	0.008	0.356
Oribacterium	*Stomatobaculum*	0.007	0.365
Stomatobaculum	*Candidatus Saccharimonas*	0.002	0.417
Tyzzerella 4	*Ruminococcaceae UCG-002*	0.041	-0.279
Tyzzerella 4	*Fusobacterium*	0.026	0.303
Ruminiclostridium 6	*Ruminococcaceae NK4A214 group*	0.010	0.349
Ruminiclostridium 6	*Ruminococcaceae UCG-002*	0.011	0.343
Ruminiclostridium 6	*Ruminococcaceae UCG-005*	0.022	0.312
Ruminiclostridium 6	*Ruminococcaceae UCG-010*	<0.001	0.542
Ruminiclostridium 6	*Thalassospira*	<0.001	0.476
Ruminococcaceae NK4A214 group	*Ruminococcaceae UCG-002*	0.019	0.317

Ruminococcaceae NK4A214 group	*Ruminococcaceae UCG-005*	<0.001	0.464
Ruminococcaceae NK4A214 group	*Ruminococcaceae UCG-010*	0.028	0.300
Ruminococcaceae NK4A214 group	*Ruminococcaceae UCG-014*	0.037	0.284
Ruminococcaceae UCG-002	*Ruminococcaceae UCG-005*	<0.001	0.527
Ruminococcaceae UCG-002	*Ruminococcaceae UCG-010*	0.047	0.272
Ruminococcaceae UCG-002	*Ruminococcaceae UCG-014*	0.042	0.277
Ruminococcaceae UCG-002	*Erysipelatoclostridium*	0.049	-0.270
Ruminococcaceae UCG-005	*Erysipelatoclostridium*	0.041	-0.280
Ruminococcaceae UCG-010	*Ruminococcaceae UCG-014*	0.005	0.377
Ruminococcaceae UCG-010	*Thalassospira*	<0.001	0.588
Ruminococcaceae UCG-010	*Candidatus Saccharimonas*	0.044	0.275
Ruminococcaceae UCG-014	*Erysipelotrichaceae Incertae Sedis*	0.027	-0.301
Erysipelatoclostridium	*Erysipelotrichaceae Incertae Sedis*	<0.001	0.558
Erysipelatoclostridium	*Megamonas*	0.048	-0.270
Erysipelatoclostridium	*Candidatus Saccharimonas*	0.036	0.287
Megamonas	*Escherichia Shigella*	0.015	-0.329

Supplementary Table 7. Correlations between plasma factors and differential genus (Type II VS. Type I)

Group	Plasma factor	Genus	*P* value	Correlation coefficient
CRC	Neutrophils	*Adlercreutzia*	0.030	-0.298
CRC	sTNFR-II	*Ruminococcaceae UCG-014*	0.046	-0.275
CRC	sTNFR-II	*Ruminococcaceae UCG-009*	0.014	-0.336
CRC	Endotoxin	*Ruminococcaceae NK4A214 group*	0.030	-0.298
CRC	Endotoxin	*Eubacterium coprostanoligenes group*	0.002	-0.410
Adenoma	Neutrophils	*Stomatobaculum*	0.011	0.343
Adenoma	sTNFR-II	*Tyzzerella 4*	0.009	0.353
Adenoma	Endotoxin	*Oxalobacter*	0.023	-0.310

Supplementary Table 8. Correlations between smoking and differential genus in type II colorectal cancer subgroup

Genus	*P* value	Correlation coefficient
Ruminiclostridium 6	0.818	-0.035
Coprococcus 2	0.916	0.016
Eubacterium ruminantium group	0.229	0.181
Cloacibacillus	0.496	0.103
Christensenellaceae R7 group	0.025	-0.331
Ruminococcaceae UCG-014	0.178	0.202
Lachnospiraceae UCG-001	0.493	0.104
Adlercreutzia	0.683	-0.062
Lachnospiraceae UCG-005	0.139	0.221
Victivallis	0.256	-0.171
Oxalobacter	0.882	0.023
Ruminococcaceae UCG-002	0.630	-0.073
Hydrogenoanaerobacterium	0.984	0.003
Ruminococcaceae UCG-010	0.273	-0.165
Ruminococcaceae NK4A214 group	0.839	0.031
Porphyromonas	0.266	-0.167
Ruminococcus 1	0.334	-0.146
Peptococcus	0.310	-0.153
Prevotella 2	0.842	0.030
Eubacterium ventriosum group	0.383	0.132
Ruminococcaceae UCG-009	0.302	-0.156
Marvinbryantia	0.218	0.185
Lachnospiraceae NK4A136 group	0.064	-0.275
Pyramidobacter	0.429	0.119
Eubacterium coprostanoligenes group	0.659	-0.067
Ruminiclostridium 9	0.350	-0.141
Lachnoclostridium	0.554	-0.090
Eggerthella	0.002	0.449
Erysipelotrichaceae incertae sedis	0.986	-0.003
Escherichia Shigella	0.551	0.09

Supplementary Table 9. Correlations between smoking and differential genus in type II colorectal adenoma subgroup

Genus	*P* value	Correlation coefficient
Thalassospira	0.958	-0.020
Ruminococcaceae UCG-010	0.958	-0.020
Ruminococcaceae UCG-014	0.349	-0.355
Lactonifactor	0.034	-0.704
Christensenellaceae R 7 group	0.944	0.028
Coprococcus 2	0.611	-0.197
Coprobacter	/	/
Ruminiclostridium 6	0.560	-0.225
Ruminococcaceae UCG-005	0.280	0.404
Holdemanella	0.351	-0.354
Ruminococcaceae UCG-002	0.403	0.319
Family XIII UCG-001	0.004	0.845
Ruminococcaceae NK4A214 group	0.479	0.272
Oxalobacter	0.947	-0.026
Adlercreutzia	0.252	-0.427
Eubacterium brachy group	0.699	-0.151
Lactococcus	0.481	0.271
Bacillus	0.925	0.037
Lachnoclostridium	0.836	0.081
Erysipelotrichaceae Incertae Sedis	0.733	-0.133
Actinomyces	0.134	0.539
Tyzzerella 4	0.947	-0.026
Fusobacterium	0.799	0.099
Erysipelatoclostridium	0.786	-0.106
Megamonas	0.179	0.491
Escherichia Shigella	0.531	0.242
Oribacterium	0.958	-0.021
Candidatus Saccharimonas	0.726	0.136
Enterococcus	0.710	-0.145
Stomatobaculum	0.731	-0.134

Supplementary Table 10. Correlations between smoking and differential genus in type I colorectal cancer subgroup

Genus	*P* value	Correlation coefficient
Ruminiclostridium 6	0.443	-0.094
Coprococcus 2	0.638	-0.058
Eubacterium ruminantium group	0.530	-0.077
Cloacibacillus	0.399	-0.103
Christensenellaceae R7 group	0.559	-0.072
Ruminococcaceae UCG-014	0.247	0.141
Lachnospiraceae UCG-001	0.586	-0.067
Adlercreutzia	0.355	0.113
Lachnospiraceae UCG-005	0.787	0.033
Victivallis	0.699	-0.047
Oxalobacter	0.585	0.067
Ruminococcaceae UCG-002	0.362	0.111
Hydrogenoanaerobacterium	0.538	0.075
Ruminococcaceae UCG-010	0.244	0.142
Ruminococcaceae NK4A214 group	0.388	-0.106
Porphyromonas	0.291	-0.129
Ruminococcus 1	0.839	0.025
Peptococcus	0.193	0.159
Prevotella 2	0.060	0.228
Eubacterium ventriosum group	0.322	0.121
Ruminococcaceae UCG-009	0.682	-0.05
Marvinbryantia	0.555	-0.072
Lachnospiraceae NK4A136 group	0.212	0.152
Pyramidobacter	0.254	-0.139
Eubacterium coprostanoligenes group	0.981	-0.003
Ruminiclostridium 9	0.592	0.066
Lachnoclostridium	0.939	0.009
Eggerthella	0.584	-0.067
Erysipelotrichaceae incertae sedis	0.508	-0.081
Escherichia Shigella	0.749	0.039

Supplementary Table 11. Correlations between smoking and differential genus in type I colorectal adenoma subgroup

Genus	*P* value	Correlation coefficient
Thalassospira	0.214	-0.155
Ruminococcaceae UCG-010	0.760	0.038
Ruminococcaceae UCG-014	0.233	-0.149
Lactonifactor	0.595	-0.067
Christensenellaceae R 7 group	0.595	0.067
Coprococcus 2	0.562	-0.073
Coprobacter	0.807	-0.031
Ruminiclostridium 6	0.801	-0.032
Ruminococcaceae UCG-005	0.385	-0.109
Holdemanella	0.864	-0.021
Ruminococcaceae UCG-002	0.320	0.124
Family XIII UCG-001	0.243	0.146
Ruminococcaceae NK4A214 group	0.711	-0.047
Oxalobacter	0.506	-0.083
Adlercreutzia	0.482	0.088
Eubacterium brachy group	0.462	-0.092
Lactococcus	0.524	0.080
Bacillus	0.403	-0.105
Lachnoclostridium	0.424	0.100
Erysipelotrichaceae Incertae Sedis	0.327	-0.122
Actinomyces	0.375	0.111
Tyzzerella 4	0.269	0.138
Fusobacterium	0.087	0.212
Erysipelatoclostridium	0.845	0.025
Megamonas	0.572	0.071
Escherichia Shigella	0.109	-0.199
Oribacterium	0.385	-0.109
Candidatus Saccharimonas	0.898	0.016
Enterococcus	0.412	-0.103
Stomatobaculum	0.469	-0.091

Supplementary Table 12. Potential microbiota markers that differentiate between the two subtypes of colorectal cancer by using linear support vector machine and logistic regression, respectively

Classifier	Threshold	AUC (mean±SD)	Biomarker	Coefficient
Linear support vector machine	0.2125	0.82±0.10	*Escherichia Shigella*	2.2751
			Eubacterium coprostanoligenes group	-0.2988
			Lachnoclostridium	0.7542
			Lachnospiraceae NK4A136 group	-0.3072
			Porphyromonas	-0.3290
			Ruminococcaceae UCG-002	-0.4211
Logistic regression	0.1698	0.84±0.10	*Escherichia Shigella*	1.0640
			Eubacterium coprostanoligenes group	-0.3273
			Lachnoclostridium	0.6098
			Lachnospiraceae NK4A136 group	-0.1934
			Porphyromonas	-0.3175
			Prevotella 2	-0.3235
			Ruminococcaceae UCG-002	-0.4762

Abbreviations: AUC, an area under the receiver operating characteristic curve; SD, standard deviation.

Supplementary Table 13. Potential microbiota markers that differentiate between the two subtypes of colorectal adenoma by using linear support vector machine and logistic regression, respectively

Classifier	Threshold	AUC (mean±SD)	Biomarker	Coefficient
Linear support vector machine	0.3459	0.86±0.11	*Escherichia Shigella*	2.3977
			Fusobacterium	1.3251
			Lachnoclostridium	1.3488
			Megamonas	2.4436
			Ruminococcaceae UCG-002	-0.5569
Logistic regression	0.2133	0.86±0.11	*Escherichia Shigella*	1.3873
			Fusobacterium	0.6964
			Lachnoclostridium	0.7799
			Megamonas	1.3853
			Ruminococcaceae UCG-002	-0.4091

Abbreviations: AUC, an area under the receiver operating characteristic curve; SD, standard deviation.

Supplementary Table 14. Detailed information on the dysregulated pathways in type II colorectal neoplasms (Type II VS. Type I)

Group	ko	Pathway	*P* value	FDR [a]	Log_2 (Fold Change)
CRC	ko04975	Fat digestion and absorption	<0.001	<0.001	-1.226
CRC	ko04666	Fc gamma R-mediated phagocytosis	<0.001	<0.001	-1.068
CRC	ko00601	Glycosphingolipid biosynthesis - lacto and neolacto series	<0.001	<0.001	-0.634
CRC	ko04622	RIG-I-like receptor signaling pathway	<0.001	<0.001	0.641
CRC	ko05100	Bacterial invasion of epithelial cells	0.020	0.048	0.652
CRC	ko00984	Steroid degradation	0.002	0.007	0.705
CRC	ko04144	Endocytosis	0.006	0.017	0.745
CRC	ko04912	GnRH signaling pathway	0.006	0.017	0.745
CRC	ko00592	alpha-Linolenic acid metabolism	<0.001	<0.001	0.860
CRC	ko01053	Biosynthesis of siderophore group nonribosomal peptides	<0.001	<0.001	0.899
CRC	ko00232	Caffeine metabolism	<0.001	<0.001	1.040
CRC	ko05204	Chemical carcinogenesis	<0.001	<0.001	1.207
CRC	ko04725	Cholinergic synapse	0.017	0.044	1.232
CRC	ko04115	p53 signaling pathway	0.004	0.013	1.512
CRC	ko04610	Complement and coagulation cascades	0.002	0.007	1.734
adenoma	ko00513	N-Glycan biosynthesis	0.011	0.027	-6.779
adenoma	ko04975	Fat digestion and absorption	<0.001	<0.001	-2.843
adenoma	ko04666	Fc gamma R-mediated phagocytosis	<0.001	<0.001	-1.621
adenoma	ko00601	alpha-Linolenic acid metabolism	<0.001	<0.001	-0.815

adenoma	ko00943	Flavonoid biosynthesis	<0.001	<0.001	-0.651
adenoma	ko04115	p53 signaling pathway	0.021	0.045	0.587
adenoma	ko00440	beta-Alanine metabolism	<0.001	<0.001	0.688
adenoma	ko00364	Benzoate degradation	<0.001	<0.001	0.747
adenoma	ko03050	Proteasome	<0.001	<0.001	0.770
adenoma	ko00592	Arachidonic acid metabolism	0.002	0.006	0.886
adenoma	ko04144	Endocytosis	0.012	0.028	0.888
adenoma	ko04912	GnRH signaling pathway	0.012	0.028	0.888
adenoma	ko00633	Glyoxylate and dicarboxylate metabolism	<0.001	<0.001	0.925
adenoma	ko04622	RIG-I-like receptor signaling pathway	<0.001	<0.001	1.094
adenoma	ko00472	Cyanoamino acid metabolism	0.007	0.018	1.094
adenoma	ko00232	Caffeine metabolism	0.001	0.003	1.101
adenoma	ko05204	Chemical carcinogenesis	0.010	0.025	1.119
adenoma	ko04610	Complement and coagulation cascades	0.002	0.006	1.547
adenoma	ko00331	Arginine and proline metabolism	<0.001	<0.001	1.868

Abbreviations: CRC, colorectal cancer; FDR, false discovery rate.
[a] FDR refers to an adjusted P value corrected by a false discovery rate.

Supplementary Table 15. Detailed information on the dysregulated genes in type II colorectal neoplasms (Type II VS. Type I)

Group	KO	Gene	*P* value	FDR [a]	Log_2 (Fold Change)
CRC	K04790	mbtC	<0.001	<0.001	-1.102
CRC	K16051	tesI	0.001	0.007	-0.940
CRC	K04782	pchB	<0.001	<0.001	-0.700
CRC	K03333	choD	<0.001	<0.001	-0.690
CRC	K04781	mbtI, irp9, ybtS	<0.001	<0.001	-0.676
CRC	K04792	mbtF	0.001	0.007	-0.389
CRC	K04784	irp2, HMWP2	0.010	0.038	-0.363
CRC	K04564	SOD2	0.005	0.022	0.224
CRC	K05296	E1.1.1.51	<0.001	<0.001	0.836
CRC	K16050	hsaD	<0.001	<0.001	0.847
CRC	K15982	kshA	0.001	0.007	0.886
CRC	K03781	katE, CAT, catB, srpA	<0.001	<0.001	0.927
CRC	K00412	CYTB, petB	<0.001	<0.001	0.958
CRC	K00863	DAK, TKFC	<0.001	<0.001	0.989
CRC	K16048	hsaB	0.006	0.025	1.204
CRC	K09483	quiC	0.005	0.022	1.217
CRC	K16047	hsaA	0.002	0.012	1.218
CRC	K01058	pldA	<0.001	<0.001	1.227
CRC	K04097	HPGDS	<0.001	<0.001	1.243

CRC	K00413	CYC1, CYT1, petC	<0.001	<0.001	1.298
CRC	K08738	CYC	<0.001	<0.001	1.373
CRC	K00632	fadA, fadI	0.001	0.007	1.433
CRC	K00365	uaZ	<0.001	<0.001	1.437
CRC	K15983	kshB	<0.001	<0.001	1.463
CRC	K00492	tmuM	<0.001	<0.001	1.486
CRC	K00799	GST, gst	<0.001	<0.001	1.518
CRC	K01253	EPHX1	<0.001	<0.001	1.553
CRC	K15653	mxcG	<0.001	<0.001	1.555
CRC	K01851	pchA	0.009	0.035	1.579
CRC	K12238	pchD	0.005	0.022	1.588
CRC	K12241	pchG	0.006	0.025	1.609
CRC	K01115	PLD1_2	0.003	0.016	1.681
CRC	K04787	mbtA	0.001	0.007	1.682
CRC	K01014	SULT1A	0.001	0.007	1.708
CRC	K00355	NQO1	<0.001	<0.001	1.709
CRC	K13285	sipB, ipaB, bipB	0.004	0.020	1.743
CRC	K13287	sipD, ipaD, bipD	0.005	0.022	1.748
CRC	K13742	ipgB1	0.005	0.022	1.749
CRC	K13286	sipC, ipaC, bipC	0.005	0.022	1.750
CRC	K01252	entB, dhbB, vibB, mxcF	0.001	0.007	1.754

CRC	K00216	entA	0.001	0.007	1.758
CRC	K13741	sopE	0.005	0.022	1.760
CRC	K02362	entD	0.002	0.012	1.763
CRC	K12785	espG, virA	0.006	0.025	1.770
CRC	K13085	ipgD, sopB	0.005	0.022	1.774
CRC	K13284	sipA, ipaA	0.005	0.022	1.775
CRC	K13743	ipgB2	0.005	0.022	1.778
CRC	K04785	irp3, ybtU	0.003	0.016	1.781
CRC	K13739	sopD	0.006	0.025	1.781
CRC	K13740	sptP	0.006	0.025	1.783
CRC	K02552	menF	<0.001	<0.001	1.829
CRC	K00510	HMOX1	<0.001	<0.001	1.848
CRC	K15981	CYP125A	0.001	0.007	2.769
CRC	K16046	CYP142	0.013	0.048	3.982
CRC	K00622	nat	0.008	0.032	4.591
CRC	K11987	PTGS2, COX2	0.013	0.048	6.239
adenoma	K00729	ALG5	<0.001	<0.001	-2.688
adenoma	K04110	badA	<0.001	<0.001	-1.876
adenoma	K16173	acd	<0.001	<0.001	-1.862
adenoma	K15372	toa	<0.001	<0.001	-1.259
adenoma	K05350	bglB	<0.001	<0.001	-1.124

adenoma	K00232	ACOX1/3	0.011	0.045	-0.974
adenoma	K07151	STT3	<0.001	<0.001	-0.942
adenoma	K00284	GLU	<0.001	<0.001	-0.806
adenoma	K03420	psmR	<0.001	<0.001	-0.793
adenoma	K04115	bcrD	<0.001	<0.001	-0.772
adenoma	K01501	E3.5.5.1	0.002	0.010	-0.751
adenoma	K09251	patA	<0.001	<0.001	-0.751
adenoma	K07130	kynB	<0.001	<0.001	-0.750
adenoma	K09123	lhpI	<0.001	<0.001	-0.738
adenoma	K13527	mpa	0.003	0.014	-0.720
adenoma	K04114	bcrA	<0.001	<0.001	-0.620
adenoma	K01432	AFMID	<0.001	<0.001	-0.616
adenoma	K01607	pcaC	<0.001	<0.001	-0.561
adenoma	K01580	gadB, gadA, GAD	<0.001	<0.001	-0.527
adenoma	K03780	ttdB	0.002	0.010	-0.527
adenoma	K03779	ttdA	0.003	0.014	-0.506
adenoma	K07535	badH	0.001	0.005	-0.487
adenoma	K11263	bccA, pccA	<0.001	<0.001	-0.486
adenoma	K07250	gabT	0.001	0.005	-0.481
adenoma	K00074	paaH, hbd, fadB, mmgB	<0.001	<0.001	-0.435
adenoma	K01464	DPYS, dht, hydA	0.001	0.005	-0.386

adenoma	K05349	bglX	0.005	0.022	-0.353
adenoma	K00248	ACADS, bcd	<0.001	<0.001	-0.337
adenoma	K00274	MAO, aofH	0.007	0.030	-0.326
adenoma	K01091	gph	<0.001	<0.001	-0.321
adenoma	K01895	ACSS1_2, acs	0.006	0.026	-0.308
adenoma	K10536	aguA	0.008	0.034	-0.287
adenoma	K00721	DPM1	<0.001	<0.001	-0.274
adenoma	K04565	SOD1	0.007	0.030	-0.258
adenoma	K01915	glnA, GLUL	<0.001	<0.001	-0.230
adenoma	K00147	proA	0.001	0.005	-0.153
adenoma	K01424	ansA, ansB	0.001	0.005	0.186
adenoma	K01458	hutG	0.005	0.022	0.336
adenoma	K00819	rocD, OAT	0.002	0.010	0.422
adenoma	K01666	mhpE	0.005	0.022	0.528
adenoma	K05606	MCEE, epi	0.005	0.022	0.538
adenoma	K07248	aldA	<0.001	<0.001	0.561
adenoma	K01847	MUT	0.006	0.026	0.586
adenoma	K00865	glxK, garK	<0.001	<0.001	0.588
adenoma	K01485	codA	0.002	0.010	0.636
adenoma	K13821	putA	0.008	0.034	0.678
adenoma	K01821	praC, xylH	0.007	0.030	0.711

adenoma	K03781	katE, CAT, catB, srpA	0.002	0.010	0.725
adenoma	K00023	phbB	0.001	0.005	0.737
adenoma	K00217	E1.3.1.32	0.002	0.010	0.800
adenoma	K00123	fdoG, fdhF, fdwA	0.007	0.030	0.847
adenoma	K10220	ligJ	0.009	0.037	0.921
adenoma	K13051	ASRGL1, iaaA	<0.001	<0.001	0.926
adenoma	K01578	MLYCD	0.006	0.026	0.926
adenoma	K10221	ligI	<0.001	0.000	0.938
adenoma	K00015	gyaR, GOR1	<0.001	<0.001	0.939
adenoma	K00412	CYTB, petB	0.001	0.005	0.963
adenoma	K01617	dmpH, xylI, nahK	0.003	0.014	0.969
adenoma	K10217	dmpC, xylG, praB	0.005	0.022	1.020
adenoma	K01055	pcaD	0.005	0.022	1.074
adenoma	K03343	puo	0.010	0.041	1.103
adenoma	K08738	CYC	0.001	0.005	1.110
adenoma	K01816	hyi, gip	<0.001	<0.001	1.123
adenoma	K01014	SULT1A	0.001	0.005	1.153
adenoma	K14449	mch, mcd	0.007	0.030	1.171
adenoma	K05783	benD-xylL	0.007	0.030	1.176
adenoma	K08692	mtkB	0.006	0.026	1.208
adenoma	K00124	fdoH, fdsB	0.003	0.014	1.210

adenoma	K14067	mtkA	0.006	0.026	1.211
adenoma	K08691	mcl	0.006	0.026	1.229
adenoma	K14447	ecm	0.006	0.026	1.235
adenoma	K00126	fdsD	<0.001	<0.001	1.236
adenoma	K00588	E2.1.1.104	0.001	0.005	1.237
adenoma	K00824	dat	0.005	0.022	1.262
adenoma	K01031	pcaI	0.004	0.018	1.283
adenoma	K00492	tmuM	0.002	0.010	1.306
adenoma	K00090	ghrB	0.001	0.005	1.337
adenoma	K01032	pcaJ	0.004	0.018	1.338
adenoma	K00632	fadA, fadI	0.001	0.005	1.352
adenoma	K00446	dmpB, xylE	0.001	0.005	1.365
adenoma	K14446	ccr	0.001	0.005	1.376
adenoma	K04097	HPGDS	<0.001	<0.001	1.398
adenoma	K00316	spdH	0.008	0.034	1.403
adenoma	K01058	pldA	<0.001	<0.001	1.411
adenoma	K00129	ALDH3	0.001	0.005	1.418
adenoma	K00355	NQO1	0.001	0.005	1.429
adenoma	K14727	pcaL	<0.001	<0.001	1.430
adenoma	K00365	uaZ	<0.001	<0.001	1.439
adenoma	K01253	EPHX1	<0.001	<0.001	1.460

adenoma	K01584	adiA	0.009	0.037	1.473
adenoma	K01577	oxc	0.001	0.005	1.476
adenoma	K02554	mhpD	0.002	0.010	1.513
adenoma	K00413	CYC1, CYT1, petC	<0.001	<0.001	1.517
adenoma	K00127	fdoI, fdsG	0.001	0.005	1.522
adenoma	K00799	GST, gst	<0.001	<0.001	1.533
adenoma	K01782	fadJ	<0.001	<0.001	1.541
adenoma	K00613	GATM	<0.001	<0.001	1.549
adenoma	K01638	aceB, glcB	0.002	0.010	1.564
adenoma	K00276	AOC3, AOC2, tynA	<0.001	<0.001	1.608
adenoma	K00448	pcaG	<0.001	<0.001	1.609
adenoma	K00137	prr	<0.001	<0.001	1.618
adenoma	K01682	acnB	<0.001	<0.001	1.639
adenoma	K00813	aspC	<0.001	<0.001	1.642
adenoma	K09471	puuB, ordL	<0.001	<0.001	1.648
adenoma	K09473	puuD	<0.001	<0.001	1.650
adenoma	K01115	PLD1_2	0.002	0.010	1.654
adenoma	K09470	puuA	<0.001	<0.001	1.658
adenoma	K01637	aceA	0.001	0.005	1.669
adenoma	K00840	astC	<0.001	<0.001	1.675
adenoma	K12972	ghrA	<0.001	<0.001	1.684

adenoma	K01608	gcl	<0.001	<0.001	1.695
adenoma	K06447	astD	<0.001	<0.001	1.700
adenoma	K01484	astB	<0.001	<0.001	1.702
adenoma	K11472	glcE	<0.001	<0.001	1.716
adenoma	K05526	astE	<0.001	<0.001	1.719
adenoma	K00673	astA	<0.001	<0.001	1.720
adenoma	K01825	fadB	<0.001	<0.001	1.724
adenoma	K00822	E2.6.1.18	<0.001	<0.001	1.732
adenoma	K03464	catC	<0.001	<0.001	1.766
adenoma	K00449	pcaH	<0.001	<0.001	1.777
adenoma	K12256	spuC	0.001	0.005	1.819
adenoma	K00495	CYP71AN24	<0.001	<0.001	1.863
adenoma	K01849	mcmA2	<0.001	<0.001	1.913
adenoma	K01848	mcmA1	<0.001	<0.001	1.918
adenoma	K05784	benC-xylZ	<0.001	<0.001	1.930
adenoma	K05550	benB-xylY	<0.001	<0.001	1.931
adenoma	K11473	glcF	<0.001	<0.001	1.938
adenoma	K05549	benA-xylX	<0.001	<0.001	1.947
adenoma	K01075	E3.1.2.23	<0.001	<0.001	1.970
adenoma	K12252	aruH	<0.001	<0.001	2.067
adenoma	K07823	pcaF	<0.001	<0.001	2.071

adenoma	K10815	hcnB	<0.001	<0.001	2.078
adenoma	K12254	kauB	<0.001	<0.001	2.146
adenoma	K12255	gbuA	<0.001	<0.001	2.158
adenoma	K12253	aruI	<0.001	<0.001	2.184
adenoma	K10814	hcnA	<0.001	<0.001	2.194
adenoma	K10816	hcnC	<0.001	<0.001	2.196
adenoma	K00460	ALOX15	<0.001	<0.001	2.214
adenoma	K04835	mal	<0.001	<0.001	2.373
adenoma	K10219	ligC	<0.001	<0.001	2.384
adenoma	K01846	glmS	<0.001	<0.001	2.940

Abbreviations: CRC, colorectal cancer; FDR, false discovery rate.
[a] FDR refers to an adjusted P value corrected by a false discovery rate.

Supplementary Table 16. Correlations between differential genes and differential genus (Type II VS. Type I)

Group	KO gene (name)	Genus	*P* value	Correlation coefficient
CRC	K01115 (PLD1/2)	*Escherichia Shigella*	<0.001	0.718
Adenoma	K01115 (PLD1/2)	*Escherichia Shigella*	<0.001	0.88
Adenoma	K01115 (PLD1/2)	*Lachnoclostridium*	0.004	0.385
Adenoma	K01115 (PLD1/2)	*Lactococcus*	<0.001	0.477
Adenoma	K01115 (PLD1/2)	*Bacillus*	<0.001	0.492
Adenoma	K01115 (PLD1/2)	*Enterococcus*	0.004	0.387
Adenoma	K01115 (PLD1/2)	*Ruminococcaceae UCG-014*	0.008	-0.357
CRC	K08738 (CYC)	*Ruminiclostridium 9*	0.047	0.275
CRC	K08738 (CYC)	*Lachnoclostridium*	0.042	0.280
Adenoma	K08738 (CYC)	*Escherichia Shigella*	<0.001	0.520
Adenoma	K08738 (CYC)	*Erysipelotrichaceae Incertae Sedis*	0.008	0.359
Adenoma	K08738 (CYC)	*Lachnoclostridium*	<0.001	0.809
Adenoma	K08738 (CYC)	*Lactococcus*	0.019	0.317
Adenoma	K08738 (CYC)	*Bacillus*	<0.001	0.618
CRC	K11987 (PTGS2/COX2)	*Lachnospiraceae NK4A136 group*	0.031	-0.297

Supplementary Figure

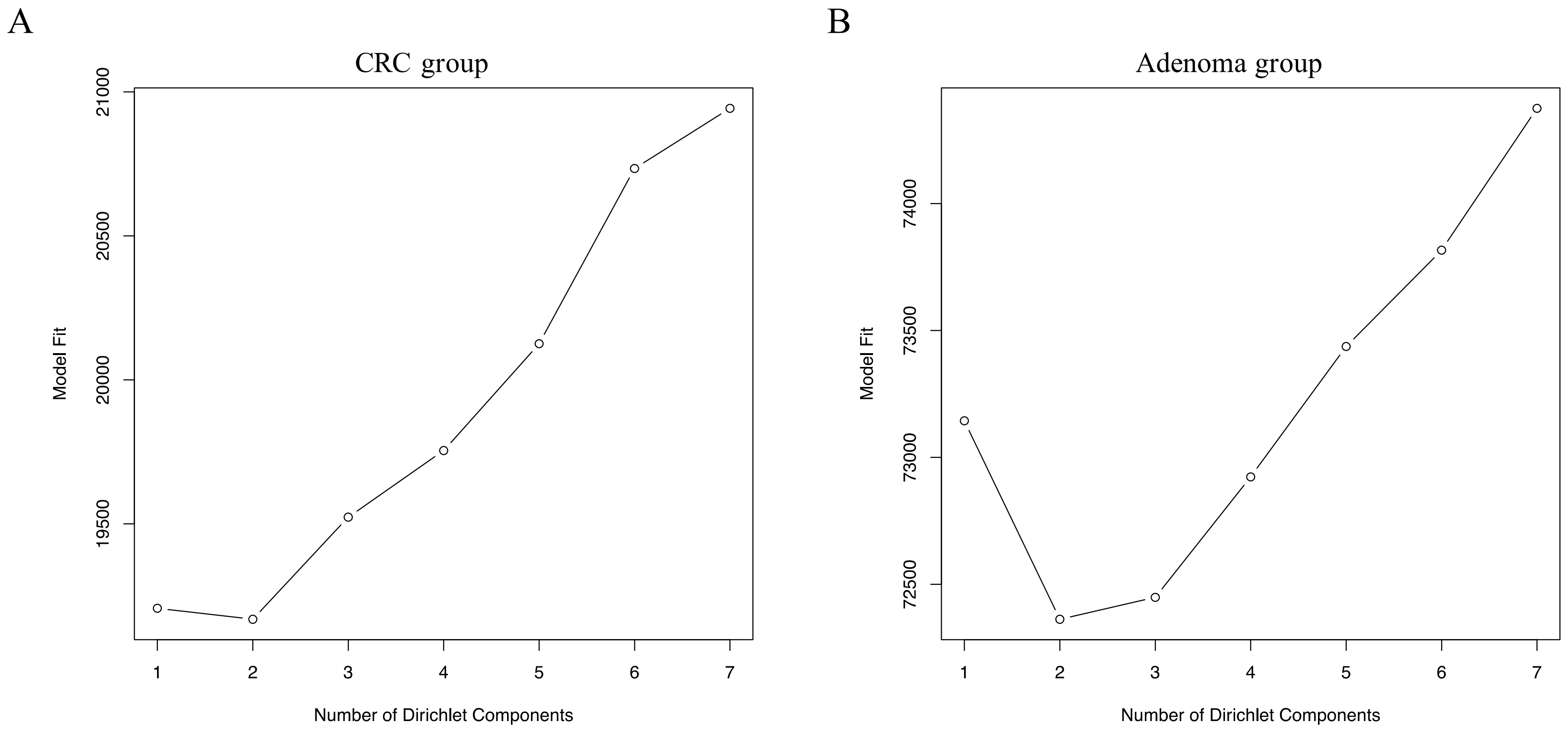

Supplementary Figure 1. Fitting classification results of Dirichlet multinomial mixture model based on gut microbiota in each case group. (A) CRC group (n = 130). (B) Adenoma group (n = 120). According to Laplace goodness of fit as the ordinate, both of the optimal fit (Laplace minimum) is for abscissa value = 2 distinct Dirichlet components.

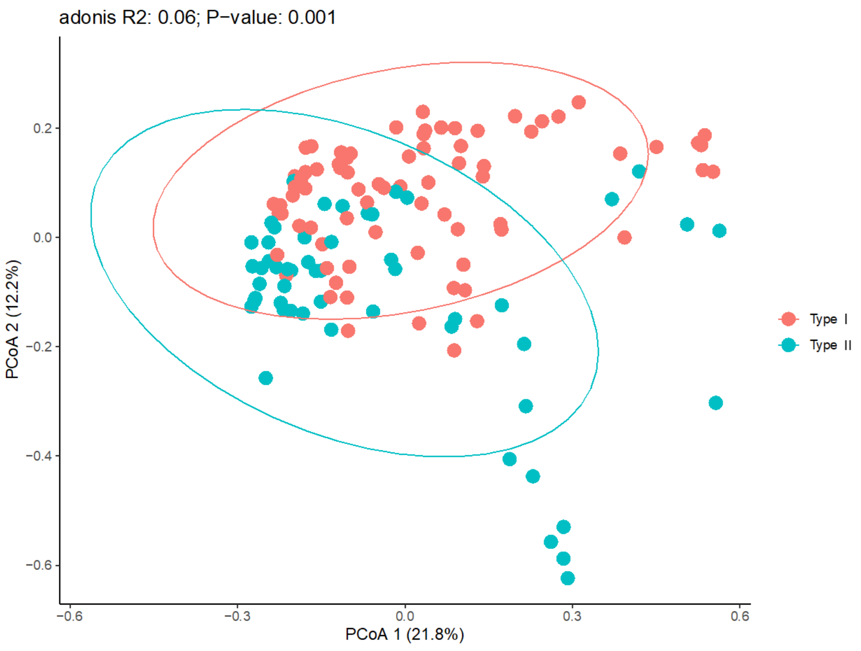

Supplementary Figure 2. Principal coordinate analysis (PCoA) plots based on Bray-Curtis distances depict the two distinct microbial community groups (type I and type II) in colorectal cancer samples. The plot displays PCoA 1 on the x-axis and PCoA 2 on the y-axis. In the plot, samples are represented by red (type I) and blue (type II) points, with surrounding ellipses illustrating the 95% confidence interval for each subtype.

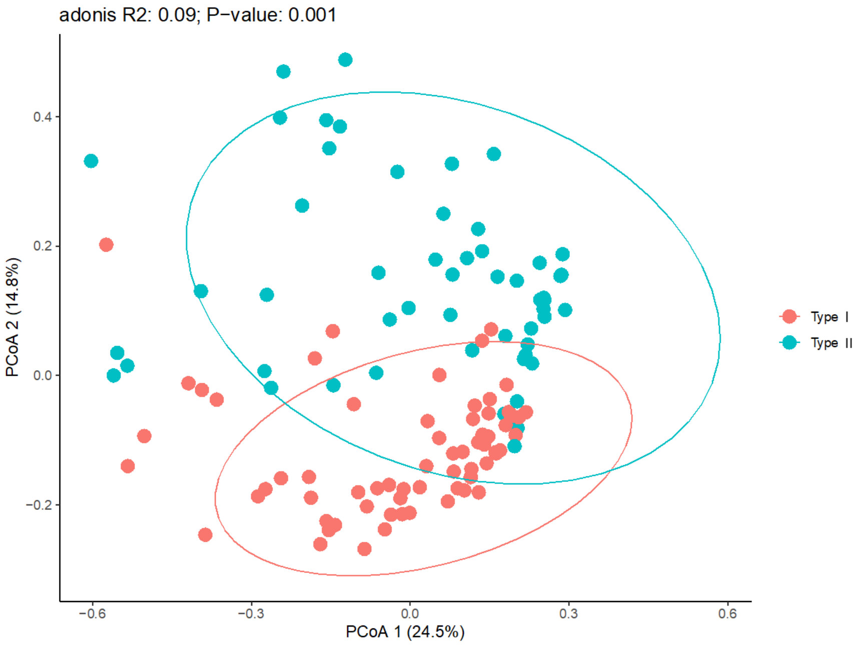

Supplementary Figure 3. Principal coordinate analysis (PCoA) plots based on Bray-Curtis distances depict the two distinct microbial community groups (type I and type II) in colorectal adenoma samples. The plot displays PCoA 1 on the x-axis and PCoA 2 on the y-axis. In the plot, samples are represented by red (type I) and blue (type II) points, with surrounding ellipses illustrating the 95% confidence interval for each subtype.

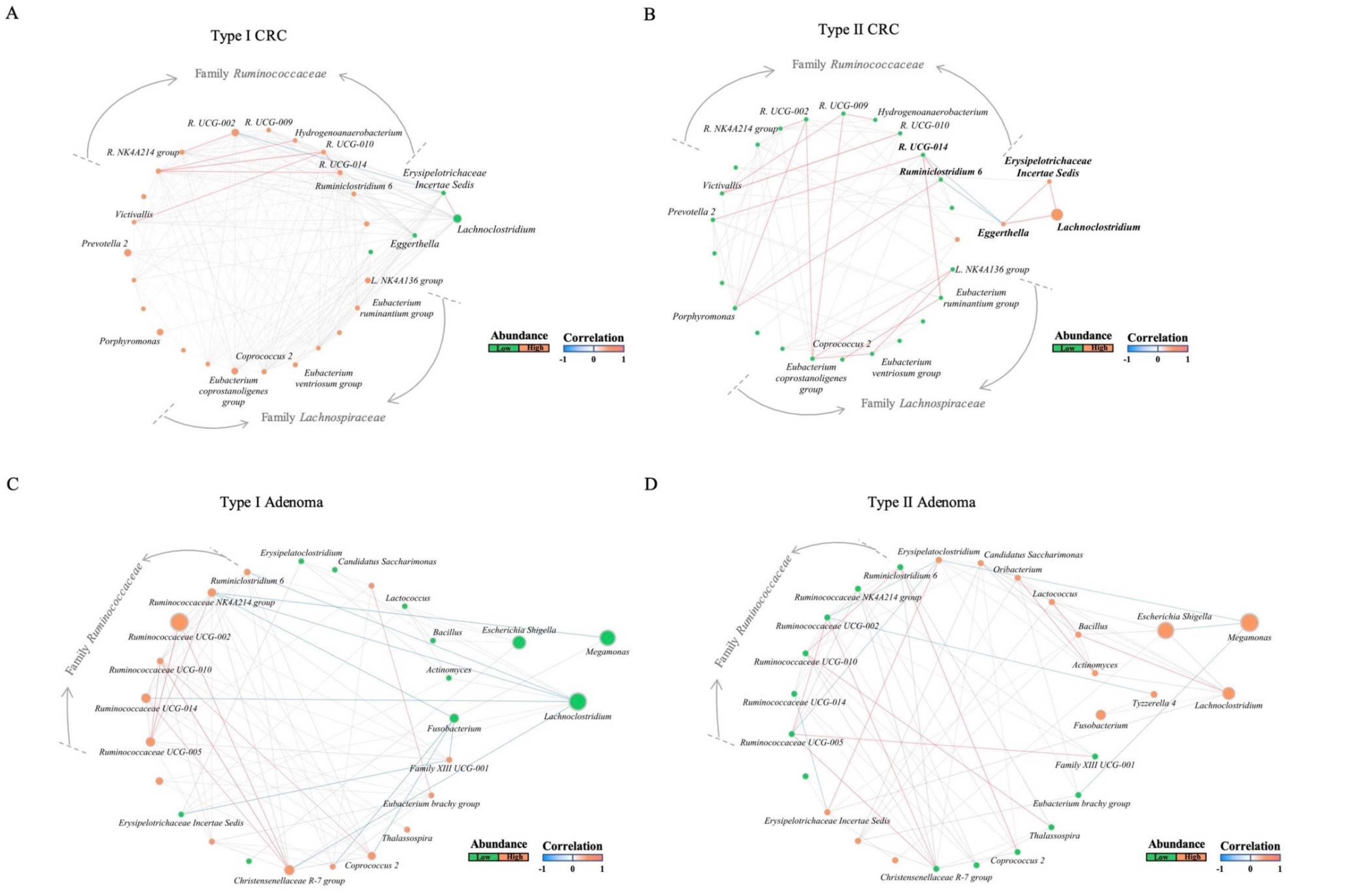
A
Type I CRC
Family Ruminococcaceae
Family Lachnospiraceae
Abundance
Low High
Correlation
-1 0 1
B
Type II CRC
C
Type I Adenoma
Family Ruminococcaceae
D
Type II Adenoma

Supplementary Figure 4. Microbial interaction networks among top 30 differential genera between type I and type II enterotypes within CRC or adenoma groups. Correlations were measured by Spearman's correlation analysis ($P < 0.05$). Node sizes are proportional to the abundance of genera, with orange representing elevated abundance and green representing decreased abundance by comparing subgroups within CRC or adenoma groups. (A) Type I CRC and (B) type II CRC. Edge color depths are proportional to correlation coefficients, with orange representing positive correlations greater than 0.4, blue representing negative correlations less than -0.45, and gray representing the rest. (C) Type I adenoma and (D) type II adenoma. Edge color depths are proportional to correlation coefficients, with orange representing positive correlations greater than 0.5, blue representing all significant negative correlations, and gray representing the rest.

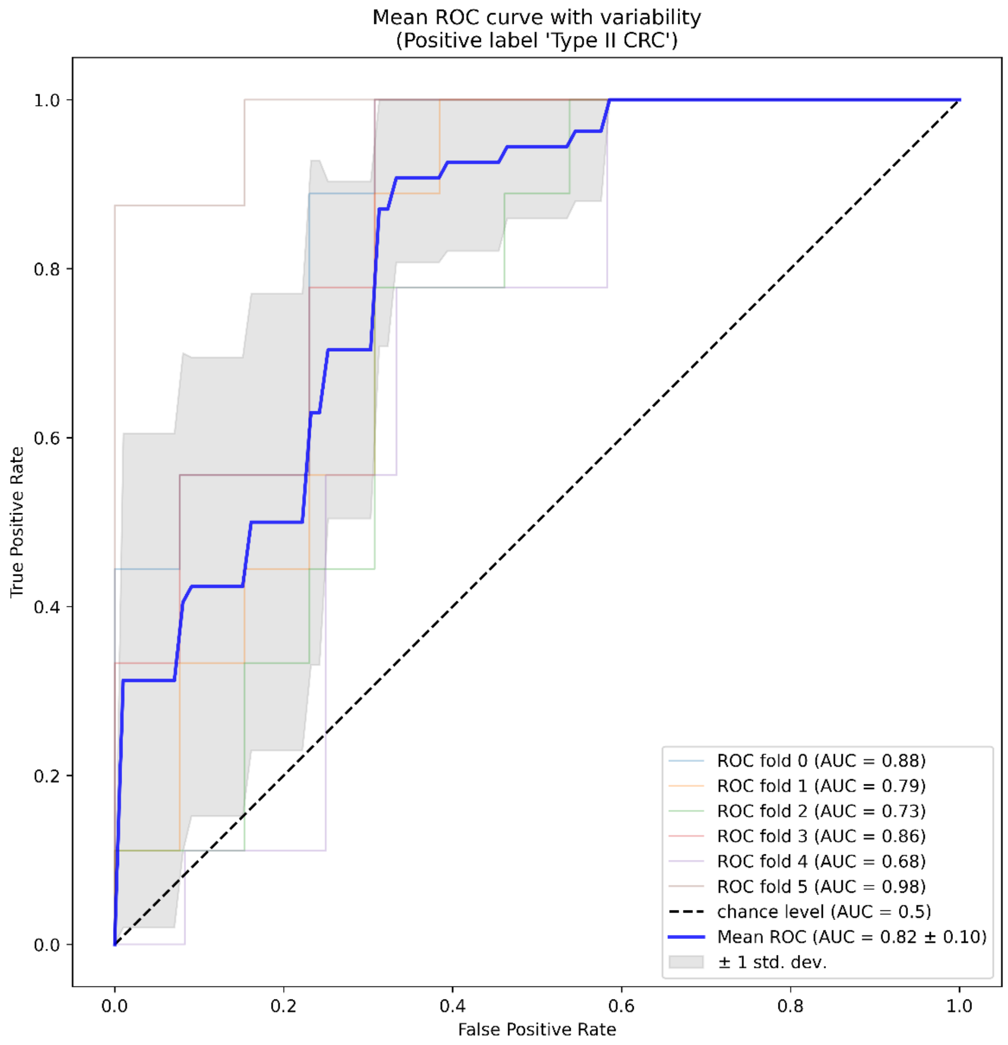

Supplementary Figure 5. Performance of the linear support vector machine classifier using AUC was evaluated using 5 randomized 5-fold cross-validation in CRC group. AUC, an area under the receiver operating characteristic curve; CRC, colorectal cancer; ROC, receiver operating characteristic.

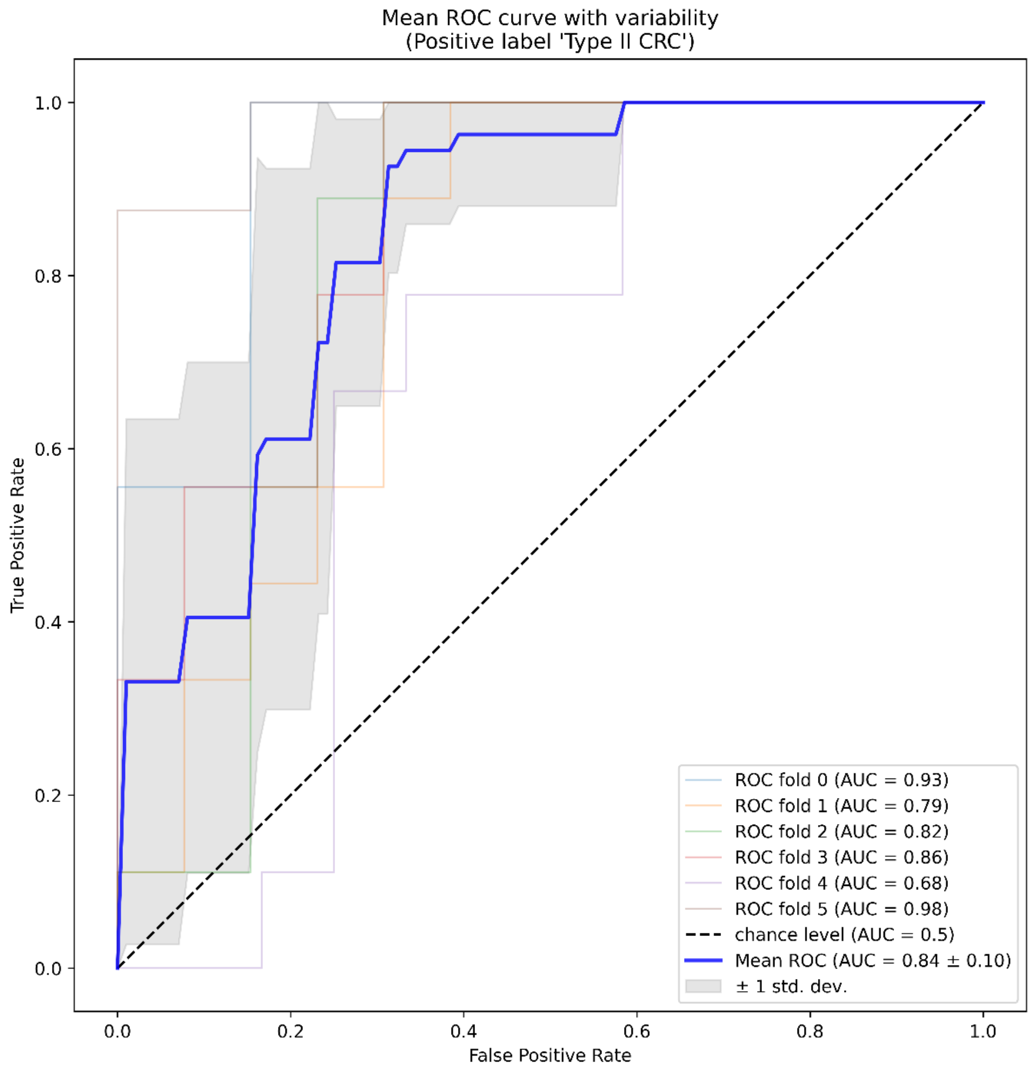

Supplementary Figure 6. Performance of the logistic regression classifier using AUC was evaluated using 5 randomized 5-fold cross-validation in CRC group. AUC, an area under the receiver operating characteristic curve; CRC, colorectal cancer; ROC, receiver operating characteristic.

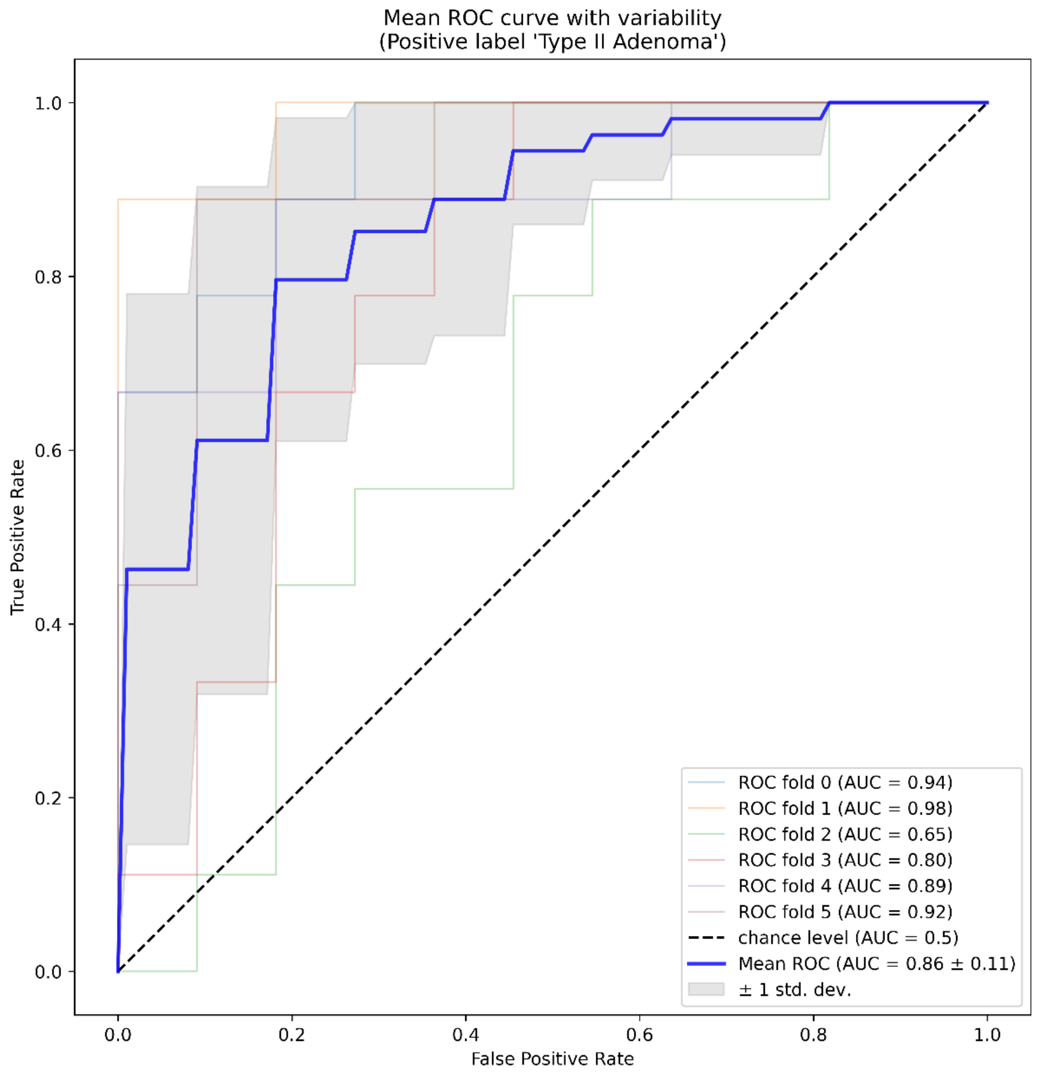

Supplementary Figure 7. Performance of the linear support vector machine classifier using AUC was evaluated using 5 randomized 5-fold cross-validation in colorectal adenoma group. AUC, an area under the receiver operating characteristic curve; ROC, receiver operating characteristic.

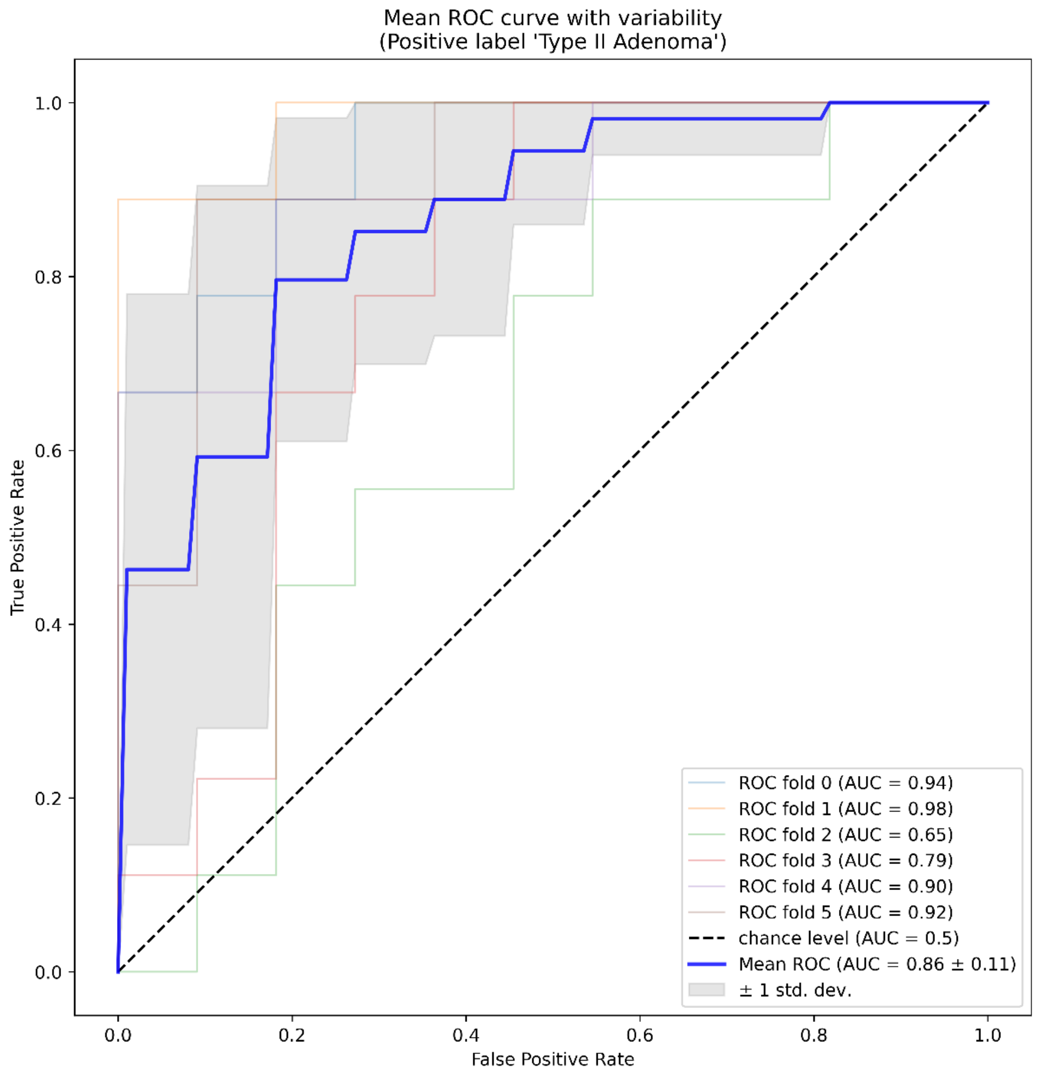

Supplementary Figure 8. Performance of the logistic regression classifier using AUC was evaluated using 5 randomized 5-fold cross-validation in colorectal adenoma group. AUC, an area under the receiver operating characteristic curve; ROC, receiver operating characteristic.

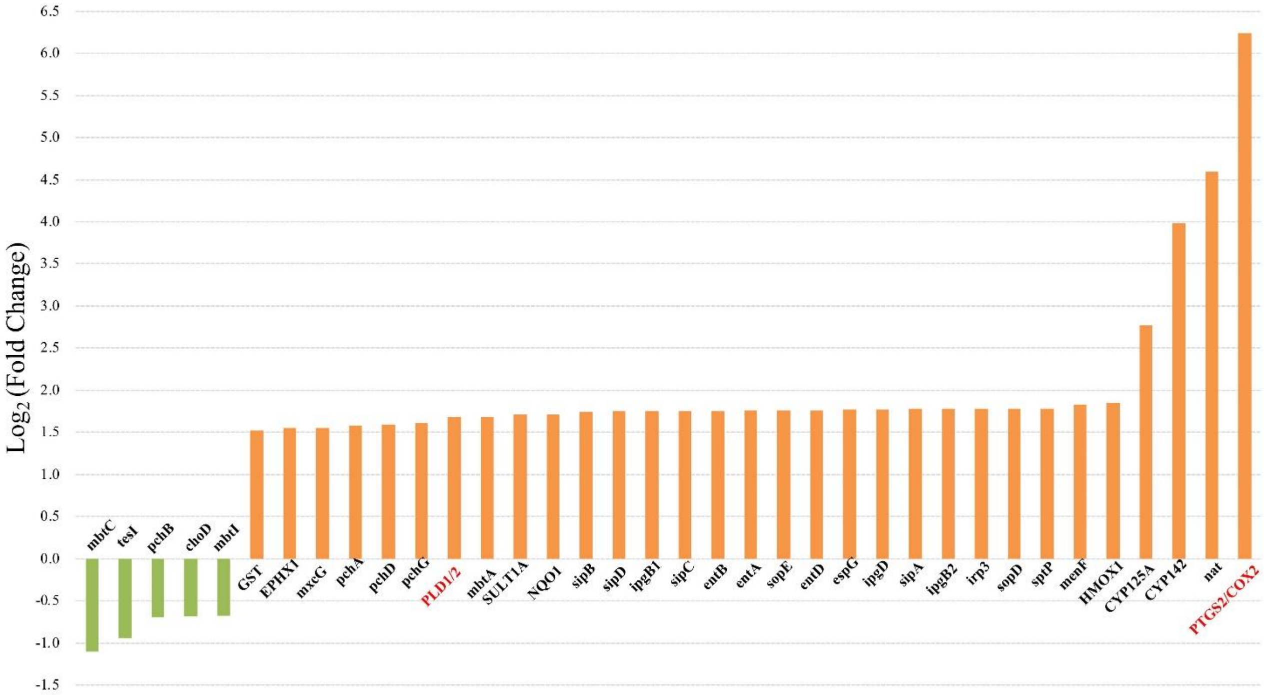

Supplementary Figure 9. Differential genes in type II CRC subtype relative to type I subtype based on Tax4FUN functional analysis (FDR adjusted p<0.05, FC>1.5). Genes that are significantly associated with differential pathways are highlighted in red. CRC, colorectal cancer; FDR, false discovery rate; FC, fold change.

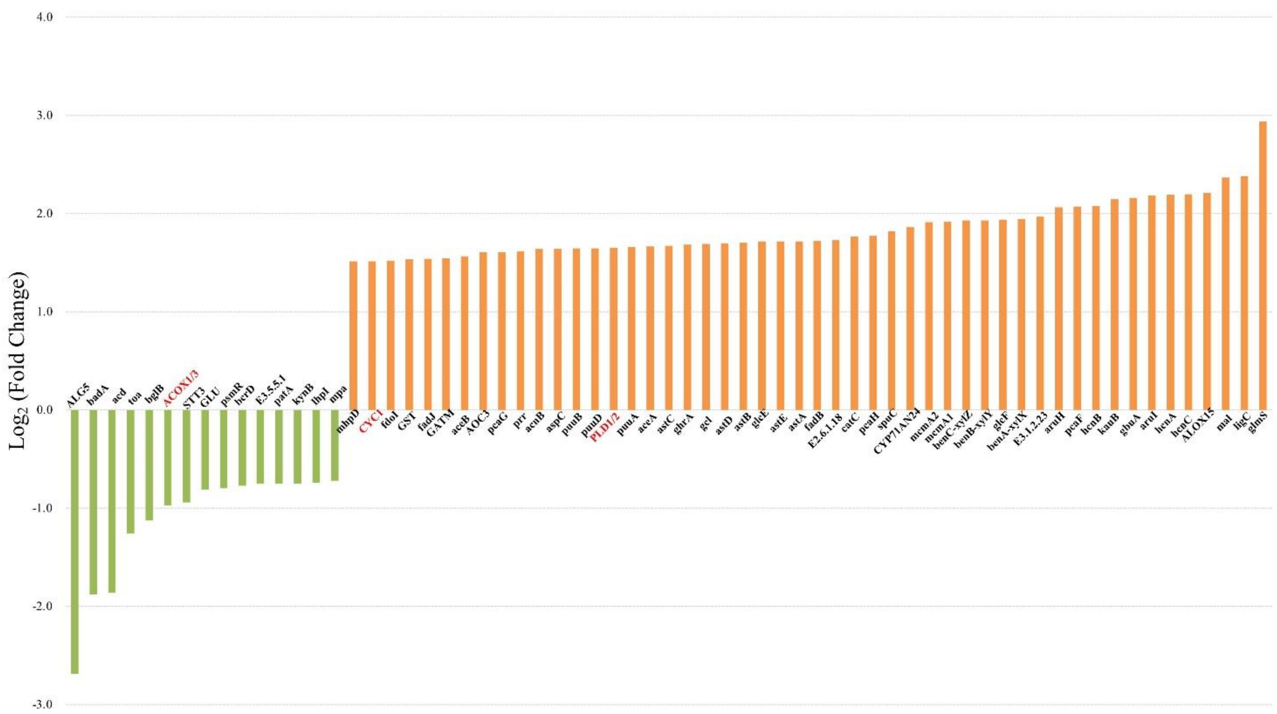

Supplementary Figure 10. Differential genes in type II colorectal adenoma subtype relative to type I subtype based on Tax4FUN functional analysis (FDR adjusted p<0.05, FC>1.5). Genes that are significantly associated with differential pathways are highlighted in red. FDR, false discovery rate; FC, fold change.

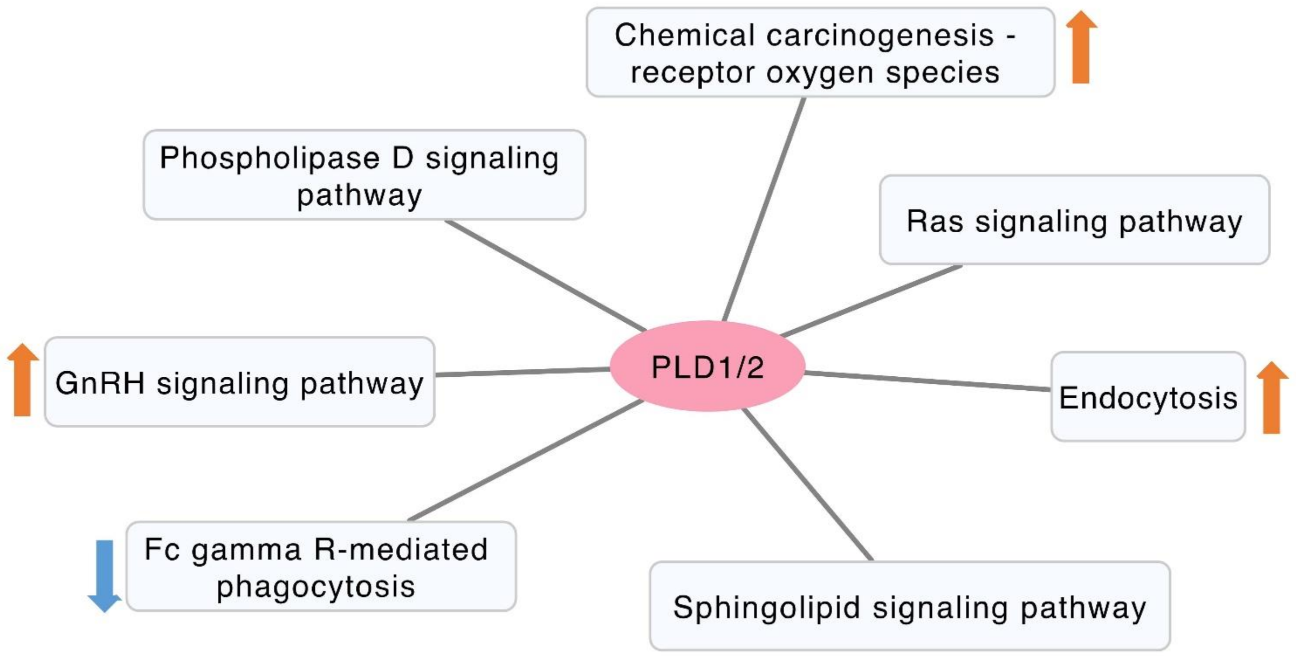

Supplementary Figure 11. The pathways involving phospholipase D 1/2 (PLD 1/2) in type II CRC subtype. The orange arrows indicate a significant increase, and the blue arrow indicates a significant decrease (FDR adjusted p<0.05, FC >1.5). CRC, colorectal cancer; FDR, false discovery rate; FC, fold change.

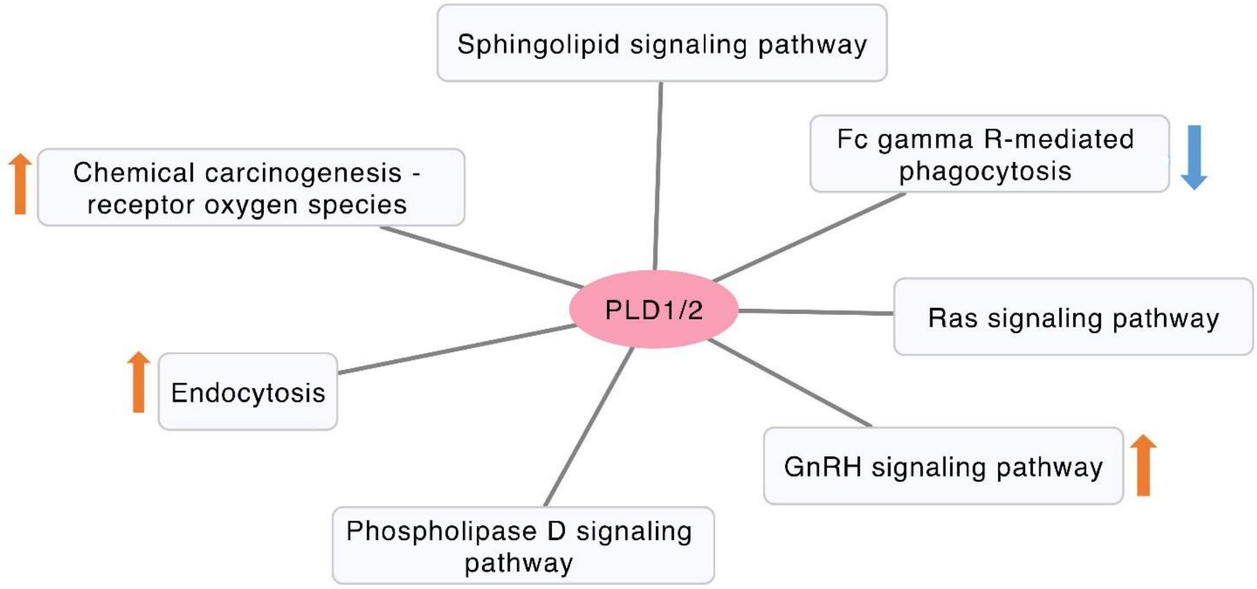

Supplementary Figure 12. The pathways involving phospholipase D 1/2 (PLD 1/2) in type II colorectal adenoma subtype. The orange arrows indicate a significant increase, and the blue arrow indicates a significant decrease (FDR adjusted p<0.05, FC >1.5). FDR, false discovery rate; FC, fold change.

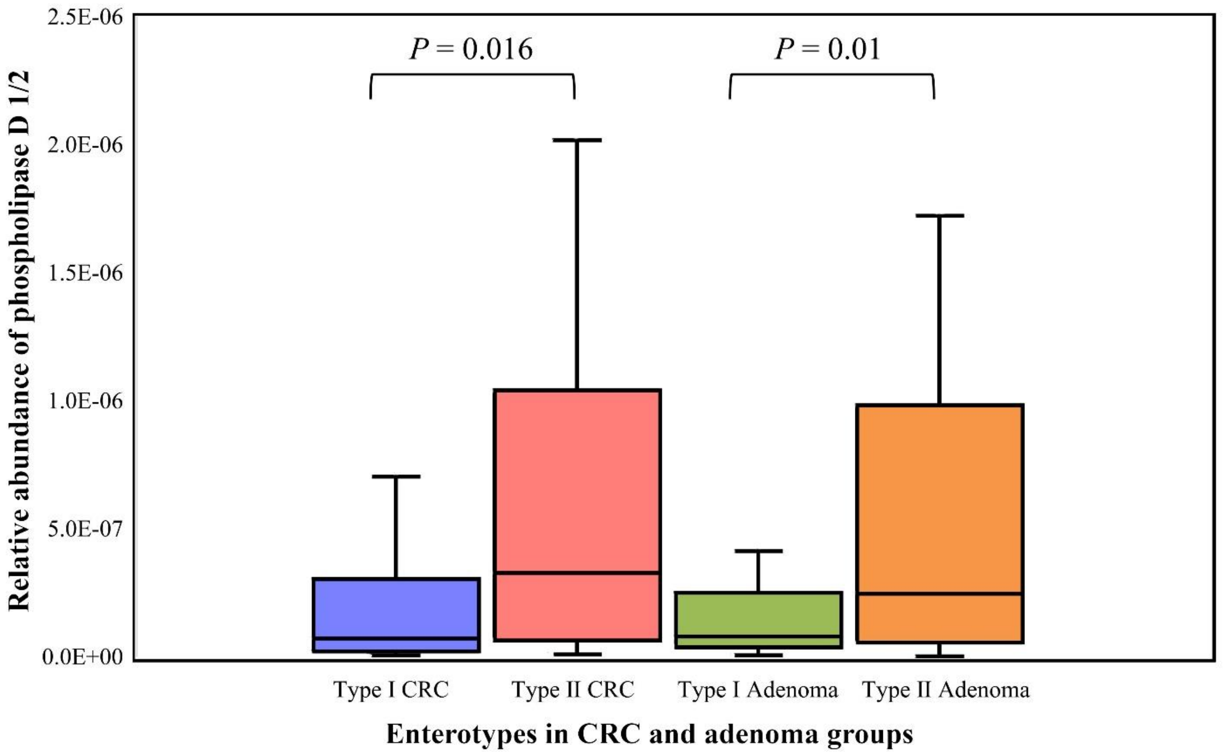

Supplementary Figure 13. The relative abundance of phospholipase D 1/2 (PLD 1/2) in different subtypes. The boxplots show the relative abundance of PLD 1/2 in type I and type II subtypes of CRC and adenoma groups. PLD 1/2 was significantly more abundant in type II subtypes than in type I subtypes in both groups (Mann-Whitney U test, all P<0.05).

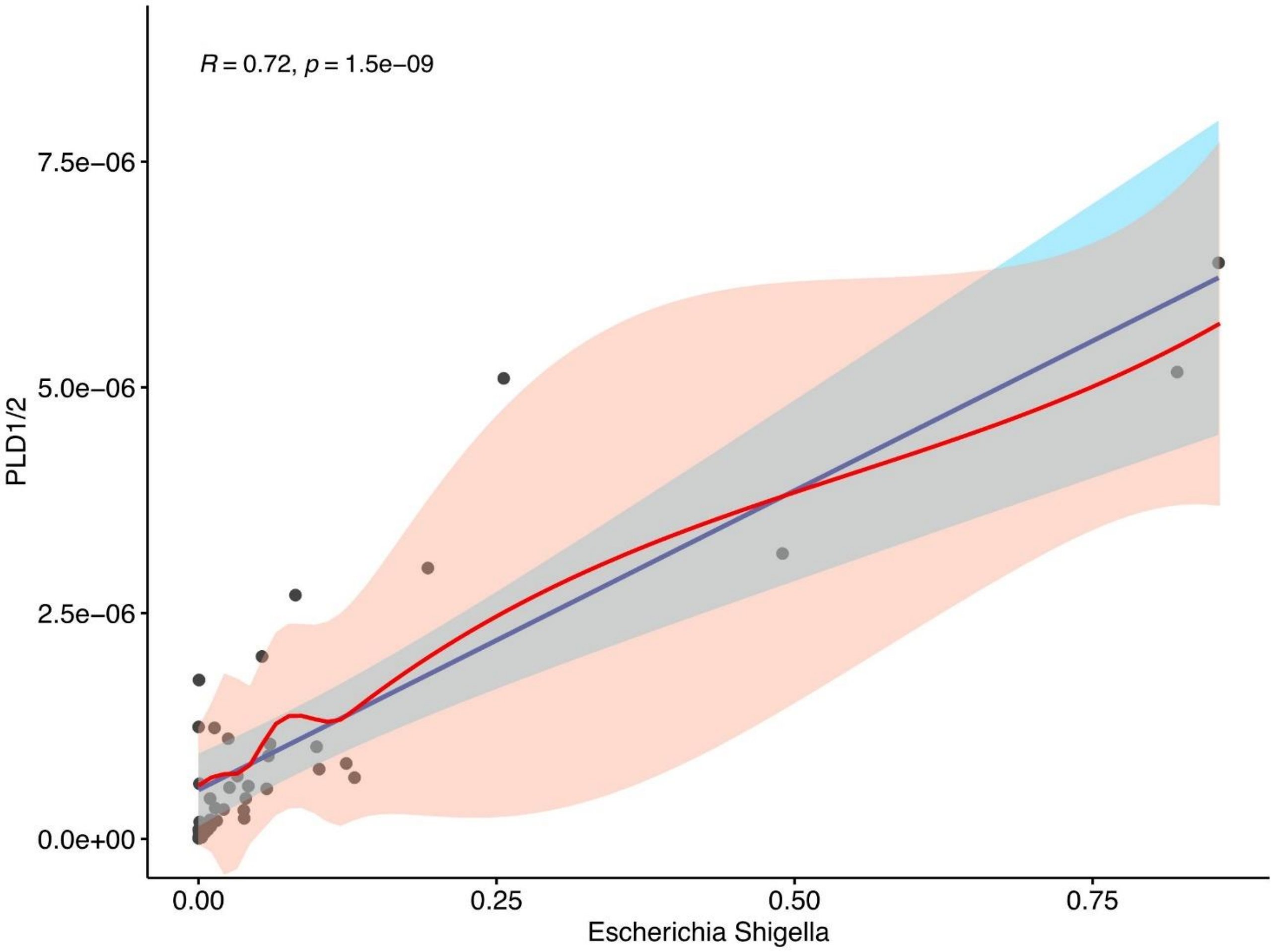

Supplementary Figure 14. Spearman's correlation analysis between the relative abundance of *Escherichia Shigella* and phospholipase D 1/2 (PLD 1/2) within type II colorectal cancer. The scatter plot shows the data points in black, the linear model fit and its 95% confidence interval in blue, and the locally weighted regression fit and its 95% confidence interval in red. The correlation coefficient R and the P value are also shown.

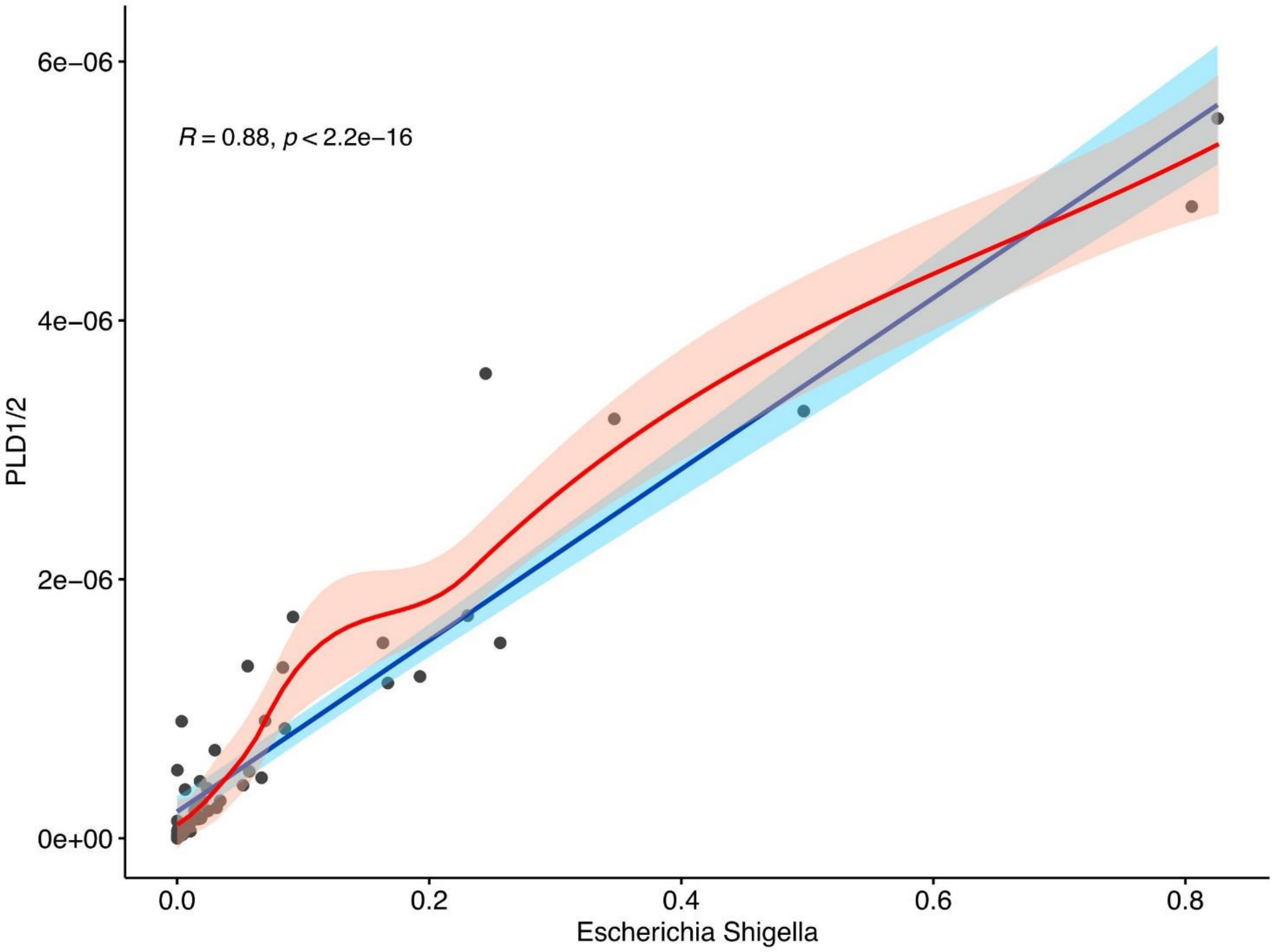

Supplementary Figure 15. Spearman's correlation analysis between the relative abundance of *Escherichia Shigella* and phospholipase D 1/2 (PLD 1/2) within type II colorectal adenoma. The scatter plot shows the data points in black, the linear model fit and its 95% confidence interval in blue, and the locally weighted regression fit and its 95% confidence interval in red. The correlation coefficient R and the P value are also shown.

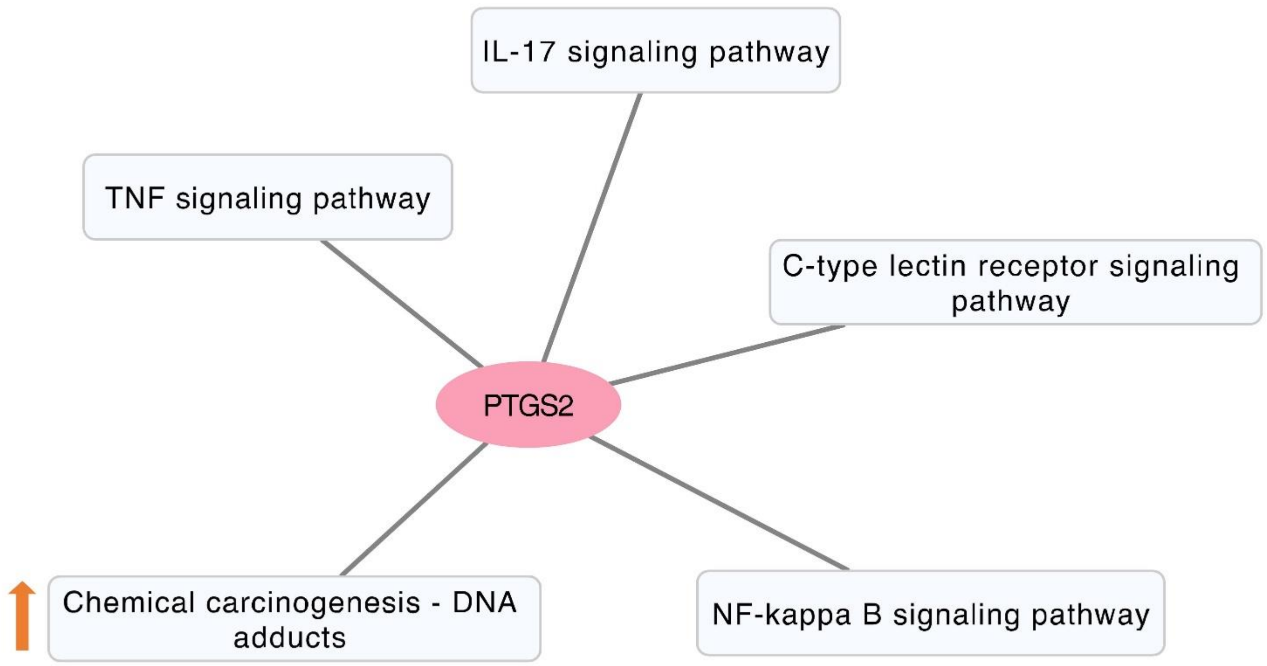

Supplementary Figure 16. The pathways involving prostaglandin-endoperoxide synthase 2 (PTGS2) in type II CRC subtype. The orange arrow indicates a significant increase (FDR adjusted $p<0.05$, FC >1.5). CRC, colorectal cancer; FDR, false discovery rate; FC, fold change.

© 2023 Cai J.A. et al.
